# ﻿Additional new species and new records of the genus *Sticta* (lichenised Ascomycota, lobarioid Peltigeraceae) from Bolivia

**DOI:** 10.3897/mycokeys.105.120810

**Published:** 2024-04-23

**Authors:** Emilia Anna Ossowska, Bibiana Moncada, Robert Lücking, Adam Flakus, Pamela Rodriguez-Flakus, Sandra Olszewska, Martin Kukwa

**Affiliations:** 1 Department of Plant Taxonomy and Nature Conservation, Faculty of Biology, University of Gdańsk, Wita Stwosza 59, PL-80-308 Gdańsk, Poland University of Gdańsk Gdańsk Poland; 2 Licenciatura en Biología, Universidad Distrital Francisco José de Caldas, Cra. 4 No. 26D-54, Torre de Laboratorios, Herbario, Bogotá D.C., Colombia Universidad Distrital Francisco José de Caldas Bogotá Colombia; 3 Research Associate, Science & Education, The Field Museum, 1400 South Lake Shore, Chicago, IL 60605, USA Research Associate, Science & Education, The Field Museum Chicago United States of America; 4 Botanischer Garten und Botanisches Museum Berlin, Freie Universität Berlin, Königin-Luise-Straße 6–8, 14195 Berlin, Germany Freie Universität Berlin Berlin Germany; 5 W. Szafer Institute of Botany, Polish Academy of Sciences, Lubicz 46, PL-31-512 Kraków, Poland W. Szafer Institute of Botany, Polish Academy of Sciences Kraków Poland; 6 10th High School in Gdynia, Władysława IV, PL-81-384 Gdynia, Poland 10th High School in Gdynia Gdynia Poland

**Keywords:** Diversity, lichens, molecular barcoding, new species, taxonomy

## Abstract

Four species of the genus *Sticta* are described as new from Bolivia, based on morphological examination and phylogenetic analysis of the fungal ITS barcoding marker. Additionally, two species are reported as new to Bolivia (their identification confirmed by molecular data) and one previously reported species is confirmed by molecular data for the first time. Detailed morphological and anatomical descriptions are provided for all new species. Two of the new species, *S.isidiolobulata* Ossowska, B. Moncada, Lücking & Kukwa and *S.madidiensis* Ossowska, B. Moncada, Lücking & Kukwa belong to clade I, as defined in previous studies. In contrast, *S.montepunkuensis* Ossowska, B. Moncada, Lücking & Kukwa and *S.macrolobata* Ossowska, B. Moncada, Lücking & Kukwa, also described here as new to science, belong to clade III. *Stictaisidiolobulata* has an irregular to suborbicular thallus of medium size, with isidia developing into spathulate lobules, cyanobacterial photobiont and apothecia with entire to weakly-crenate margins. The large irregular thallus of the cyanobacteria-associated *S.macrolobata* has broad lobes, apothecia with verrucous to tomentose margins and cyphellae with raised margins, whereas *S.madidiensis* has a medium-sized, palmate to irregular thallus with a stipe, but without vegetative propagules and apothecia. *Stictamontepunkuensis* has large and irregular thalli with green algae as photobiont, apothecia with crenate to verrucous margins and urceolate cyphellae with a wide pore and a scabrid basal membrane. Two species, *S.beauvoisii* Delise and *S.riparia* Merc.-Díaz are reported as new to Bolivia (the latter also as new to South America) and belong to clade III. *Stictatomentosa* (Sw.) Ach., species confirmed from Bolivia by molecular data, belongs to clade II. *Stictabeauvoisii* is characterised by a smooth yellowish-brown upper surface with darker apices and abundant, marginal isidia and a brown lower surface with golden-chocolate brown primary tomentum and sparse, golden-brown rhizines. *Stictariparia* has a strongly branched thallus, with undulate lobes and abundant, marginal, palmate, grey to dark brown phyllidia and greyish-brown lower surface with the primary tomentum absent towards the margins. *Stictatomentosa* has palmate, bluish thalli with white cilia and abundant, submarginal apothecia and creamy-white lower surface with a sparse, white primary tomentum.

## ﻿Introduction

The name *Sticta* was first introduced by Schreber (1791), who classified these lichens as a section within the genus *Lichen*. Later, Acharius (1803) raised *Sticta* to the rank of a genus. *Sticta* has a subcosmopolitan distribution and includes macrolichens with true cyphellae and tomentum present at least on the lower surface of the thalli, for example, Galloway (1994, 1995), Moncada (2012) and Moncada et al. (2018, 2020). At the beginning of the 21^st^ century, about 120 species were known within the genus (Kirk et al. 2008), but recently, the number of known taxa has tripled (Moncada et al. 2021a). This increase is mainly related to the application of integrative taxonomy, based on molecular, morphological and anatomical data and the exploration of tropical regions, which are often habitats of unknown, endemic species (Tønsberg and Goward 2001; Moncada 2012; Lendemer and Goffinet 2015; Moncada et al. 2018, 2020; Mercado-Díaz et al. 2020; Ossowska et al. 2022a). In Colombia, for example, intensive field surveys and laboratory analyses have increased the number of identified *Sticta* from 42 (Sipman et al. 2008) to 150 (Moncada 2012; Moncada et al. 2013a, b, 2014). Similar explorations have been carried out in other regions of the Neotropics (Dal Forno et al. 2018; Torres et al. 2021; Ossowska 2021; Ossowska et al. 2022a, b; Crous et al. 2023), as well as in other parts of the world (McDonald et al. 2003; Simon et al. 2018; Moncada et al. 2020, 2021a; Di Meglio and Goward 2023; Kaasalainen et al. 2023). However, in many regions, the genus *Sticta* is still in need of revision, so the number of described species within the genus is much lower than estimated (Moncada et al. 2021a, b, c).

In Bolivia, located in the central part of the Neotropical Region of South America, research on *Sticta* has been conducted since the 19^th^ century (Nylander 1859, 1861; Rusby 1896; Herzog 1922, 1923; Feuerer et al. 1998). As a result, eleven *Sticta* species were reported, based solely on their morphological and anatomical characters (Ossowska 2021 and literature cited therein). Recently, modern approaches in the taxonomy of *Sticta*, including molecular analyses, have been applied to Bolivian collections, resulting in recording nine additional species, including seven new to science (Moncada and Lücking 2012; Ossowska et al. 2022a, b; Crous et al. 2023). Here, we present the descriptions and records of seven additional species, including four new to science (*Stictaisidiolobulata* Ossowska, B. Moncada, Lücking & Kukwa, *S.macrolobata* Ossowska, B. Moncada, Lücking & Kukwa, *S.madidiensis* Ossowska, B. Moncada, Lücking & Kukwa and *S.montepunkuensis* Ossowska, B. Moncada, Lücking & Kukwa), two new to Bolivia (*S.beauvoisii* Delise and *S.riparia* Merc.-Díaz) and the first record of *S.tomentosa* (Sw.) Ach. confirmed by molecular data.

## ﻿Material and methods

### ﻿Taxon sampling

The study was based on specimens collected during fieldwork in the Yungas and Tucumano-Boliviano Regions of Bolivia and deposited at KRAM, LPB and UGDA Herbaria. Morphology and anatomy were examined under stereo- and compound microscopes (Nikon SMZ800N and ZEISS Axioskop). Spot test reactions were made with K (potassium hydroxide solution), C (sodium hypochlorite solution), Pd (paraphenylenediamine) and KC (K followed by C on the same thallus fragments). Secondary compounds were further analysed using thin-layer chromatography (TLC) in solvents A and C (Orange et al. 2001).

Species, which were distinguished by Moncada (2012), but have not yet been formally described are marked with quotes (e.g. ‘*S.isidioimpressula*’).

### ﻿DNA extraction, PCR amplification and sequencing

The protocols for DNA extraction and sequencing of the nuITS rDNA marker followed Ossowska et al. (2022a).

### ﻿Sequence alignment and phylogenetic analysis

The obtained sequences were aligned with available sequences of the genus *Sticta* (Suppl. material 1: table S1), using our previous alignment (Ossowska et al. 2022a) based on a recent master alignment (Moncada et al. 2020). The new sequences were added to the existing alignment using MAFFT 7.164 with the “--add” option (Katoh and Frith 2012; Katoh and Standley 2013), with subsequent manual inspection in BIOEDIT 7.0.9 (Hall 2011). Phylogenetic analysis was performed using Maximum Likelihood in RAxML 8.2.0 (Stamatakis 2014) on the CIPRES Science Gateway (Miller et al. 2010), with non-parametric bootstrapping using 400 pseudoreplicates (based on an automated saturation criterion) under the universal GTRGAMMA model. Trees were visualised in FigTree 1.4.2 (Drummond and Rambaut 2007). After initial analysis of the full taxon set containing 1,049 terminals, the alignment was reduced to a subset containing 3–10 accessions per species, for a total of 211 terminals and the phylogenetic analysis was repeated using the above approach.

## ﻿Results and discussion

**Figure 1.**
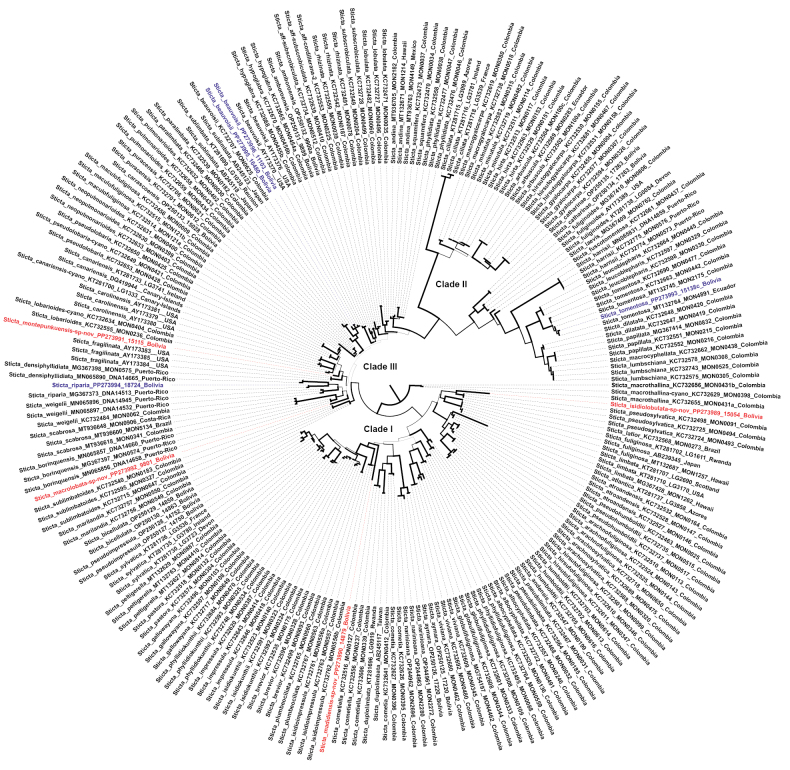
Best-scoring Maximum Likelihood tree of the *Sticta* target clade containing the new species from Bolivia (red) and the species new to Bolivia and phylogenetically confirmed from Bolivia (blue), based on the fungal ITS barcoding marker. Supported clades are thickened. For complete tree with individual support values, see Suppl. material 2.

Seven new nuITS rDNA sequences were generated for this study. Three of these clustered into clades of previously-defined *Sticta* (McDonald et al. 2003; Moncada 2012; Widhelm et al. 2018; Mercado-Díaz et al. 2020). These are *S.beauvoisii* and *S.riparia*, which are new to Bolivia, and *S.tomentosa*, which was reported from Bolivia, but not confirmed with molecular data until now (Fig. 1). Notes on all three species are given below.

Four sequences form distinct lineages, suggesting previously undescribed taxa, are grouped within clades I (*fuliginosa* clade) and III (*weigelii* clade), as defined by Widhelm et al. (2018) (Fig. 1). Comparison of morphological and anatomical features of these specimens with similar and related taxa, as well as phylogenetic analysis, confirmed they represent species new to science. These are *Stictaisidiolobulata* sp. nov. and *S.madidiensis* sp. nov. in clade I and *S.montepunkuensis* sp. nov. and *S.macrolobata* sp. nov. placed in clade III sensu Widhelm et al. (2018) (Fig. 1). Detailed descriptions of all four new *Sticta* species are given below.

At present, the genus *Sticta* contains more than 500 species and more than one hundred morphological and sixty anatomical characters can be used for their circumscriptions (Moncada et al. 2014, 2021a; Ossowska et al. 2022a; Kaasalainen et al. 2023). These include the presence and type of vegetative propagules, such as isidia, phyllidia, soredia or lobules (Galloway 1994, 1995; Moncada 2012; Moncada et al. 2014; Ossowska et al. 2022a; Kaasalainen et al. 2023). Moreover, some species may develop two types of propagules, like the newly-introduced *S.isidiolobulata*, which has isidia and lobules or *S.cyanocaperata* Kaasalainen and *S.andina* B. Moncada, Lücking & Sérus. having isidia and phyllidia (Moncada 2012; Moncada et al. 2021b; Kaasalainen et al. 2023). Of the *Sticta* species known from Bolivia with records confirmed by molecular data, seven produce vegetative propagules and most of them have isidia: *S.andina*, *S.aymara* Ossowska et al., *S.beauvoisii*, *S.isidiokunthii* B. Moncada & Lücking and *S.weigelii* (Ach.) Vain. (Moncada et al. 2014; Ossowska 2021; Ossowska et al. 2022a). The structure of the isidia and their distribution on the thallus, as well as their colour and size, are diagnostic to distinguish species. For example, in *S.isidiokunthii*, isidia are darker and in *S.beauvoisii* lighter than the thallus; they are cylindrical to squamiform in *S.beauvoisii* and globular to cylindrical in *S.aymara*, palmate to coralloid in *S.andina* and cylindrical in other isidiate species (Moncada 2012; Moncada and Lücking 2012; Moncada et al. 2021b; Ossowska 2021; Ossowska et al. 2022a).

Phyllidia resemble isidia, but are flattened and dorsiventral (Nash 2008); they are known in two taxa from Bolivia, *S.scabrosa* B. Moncada, Merc.-Díaz & Bungartz subsp. scabrosa and *S.riparia*. They are dark brown in *S.riparia*, whereas of the same colour as the thallus in *S.scabrosa* subsp. scabrosa (Moncada 2012; Mercado-Díaz et al. 2020; Moncada et al. 2021b; Ossowska et al. 2022b).

Lobules are like small lobes, i.e. forming minute cyphellae and partly also tomentum on the lower surface (Nash 2008; Moncada 2012; Mercado-Díaz et al. 2020; Moncada et al. 2020, 2021a). To date, only a few *Sticta* species with lobules have been described, for example, *S.guilartensis* Merc.-Díaz from Puerto Rico or *S.antoniana* B. Moncada & Lücking from Hawaii (Moncada 2012; Mercado-Díaz et al. 2020; Moncada et al. 2020, 2021a). *Stictaisidiolobulata* described here is the first lobulate species from Bolivia; in this taxon, the lobules develop from isidia, especially at the edges of lobes and it is one of its diagnostic features.

Soredia are the rarest type of propagules found in *Sticta* (Moncada 2012; Moncada et al. 2013b); however, none of the known sorediate taxa has been found in Bolivia so far.

Another important diagnostic feature of *Sticta* is the presence of apothecia. The fertile species in Bolivia are: *S.amboroensis* Ossowska et al., *S.bicellulata* Ossowska et al., *S.carrascoensis* Ossowska et al., *S.catharinae* Ossowska et al., *S.macrolobata*, *S.monlueckiorum* Ossowska, Flakus & Rodr.Flakus, *S.montepunkuensis*, *S.pseudoimpressula* Ossowska et al. and *S.tomentosa* (Moncada 2012; Ossowska et al. 2022a, this paper; Crous et al. 2023). The most important differences between them are the abundance of apothecia (scarce in *S.bicellulata* and *S.montepunkuensis* or abundant in *S.catharinae* and *S.amboroensis*), their distribution (submarginal in *S.tomentosa*, laminal to submarginal in *S.macrolobata* or marginal in *S.carrascoensis*) or the structure of the apothecial margins (crenate to hirsute in *S.amboroensis*, crenate to verrucose in *S.montepunkuensis*, verrucous to tomentose in *S.macrolobata*, entire to crenate in *S.pseudoimpressula*, hirsute to ciliate, but in young apothecia glabrous in *S.monlueckiorum*) (Moncada 2012; Ossowska et al. 2022a; Crous et al. 2023). In addition, there is a group of *Sticta* species that have two forms: with apothecia and vegetative propagules as in *S.andina*, the newly introduced *S.isidiolobulata* and S.scabrosasubsp.scabrosa (but only in specimens from Bolivia both, phyllidia and apothecia, are present) (Moncada et al. 2014, 2021a, b; Ossowska et al. 2022b).

The cyanobacteria-associated *Stictamadidiensis* lacks both apothecia and vegetative propagules. In *Sticta*, such situations are mostly known in species possessing two photosymbiodemes, i.e. lichen thallus can be formed with a green alga or a cyanobacteria (e.g. in *S.lobarioides* B. Moncada & Coca or *S.pseudolobaria* B. Moncada & Coca). In such cases, the green algal form has abundant apothecia, whereas the cyanobacterial form usually lacks apothecia and vegetative propagules (Moncada 2012; Moncada et al. 2013a) as it is in *S.madidiensis*. Potentially *S.madidiensis* is a species that also forms photosymbiodemes, but at present, only the cyanobacterial thalli are known.

Morphodemes, which are species that are morphologically and anatomically similar, but phylogenetically distant, are common in the genus *Sticta* (Moncada et al. 2021b). For instance, *S.andina* and *S.scabrosa* are morphodemes of *S.weigelii* (Moncada et al. 2020, 2021b), whereas *S.arenosella* Di Meglio & Goward and *S.gretae* Goward & Di Meglio are morphodemes of *S.fuliginosa* (Di Meglio and Goward 2023). Such taxa have also been found in Bolivia and *S.isidiolobulata* introduced in this paper and *S.pseudoimpressula* described by Ossowska et al. (2022a) are morphodemes of *S.impressula* (Nyl.) Zahlbr. They all have a pitted to scrobiculate or rugose upper surface with apothecia and cilia (Moncada 2012; Ossowska et al. 2022a), but differ in the structure of the cyphellae and also in the colour and thickness of the primary and secondary tomentum. In addition, *S.isidiolobulata* also produces isidia and lobules, which are absent in *S.impressula*. Despite similarities to *S.impressula*, the new *S.isidiolobulata* is closely related to the still undescribed species ‘*S.pseudosylvatica*’, whereas *S.pseudoimpressula* forms a clade with *S.bicellulata* (Ossowska et al. 2022a). *Stictamontepunkuensis* belongs to the *S.laciniata* morphodeme, which has a green algal photobiont, a scrobiculate upper surface, with apothecia and without true cilia, but with a visible extension of the lower tomentum (Hooker 1822). The differences are also in the size of the thalli (in *S.laciniata* (Sw.) Ach., the thallus is smaller and highly branched), the distribution of the apothecia (in *S.montepunkuensis*, they are mainly laminal and subaggregated and in *S.laciniata*, submarginal and dispersed), the apothecial margins (in *S.laciniata* apothecia margins are tomentose and, in *S.montepunkuensis* crenate to verrucous) and the density of the cyphellae (*S.montepunkuensis* has more abundant cyphellae towards the margins and in the centre than *S.laciniata*).

The diversity of lichen species in Bolivia is still not fully understood; however, recent results systematically increase the number of species known from this country (Flakus et al. 2019; Guzow-Krzemińska et al. 2019; Kukwa and Ossowska 2022; Kukwa et al. 2023a, b). The knowledge on *Sticta* in Bolivia is also increasing and recent morphological and anatomical studies supported by phylogenetic analyses have contributed significantly to this (Ossowska 2021; Ossowska et al. 2022a, b; Crous et al. 2023). With the taxa described here, *Sticta* currently comprises twenty-six species in Bolivia (three other recorded species are definitely misidentifications; see Ossowska et al. (2022a)), of which twenty-one are confirmed by molecular data. A further five species remain to be verified; these are *S.dilatata* (Nyl.) Vain, *S.fuliginosa* (Dicks) Ach., *S.kunthii* Hook., *S.laciniata* and *S.sinuosa* Pers. (Rodriguez-Flakus et al. (2016) and literature cited therein). Literature data suggest that at least *S.dilatata* and *S.fuliginosa* can also be present in Bolivia as they have been recorded from other South American countries (Widhelm et al. 2018). *Stictafuliginosa* s.str. is a subcosmopolitan species (McDonald et al. 2003; Hodkinson et al. 2014; Magain and Sérusiaux 2015; Di Meglio and Goward 2023; Kaasalainen et al. 2023) and, in South America, is known only from Brazil (Magain and Sérusiaux 2015; Widhelm et al. 2018). However, numerous morphodemes of *S.fuliginosa* are known from the Neotropics (Moncada et al. 2015); therefore, it is likely that Bolivian material may have represented one or several of these. It is the same with *S.laciniata.* Records of the species may belong to other species, for example, to *S.montepunkuensis*, which is described as a new species in this paper and belong to the *S.laciniata* morphodeme. The phylogenetic positions of *S.kunthii* (described from Peru) and *S.sinuosa* (described from Brazil) have not so far been confirmed by molecular data (Moncada 2012; Kaasalainen et al. 2023), so it may be difficult to prove their presence in Bolivia. *Stictakunthii* was reported from Africa by Kirika et al. (2012), but Kaasalainen et al. (2023) did not find this species amongst the studied species of *Sticta*. The authors concluded that the specimens reported by Kirika et al. (2012) probably represent *S.umbilicariiformis* Hochsc. ex Flotow and/or *S.aspratilis* Kaasalainen & Rikkinen (Kaasalainen et al. 2023).

It is worth noting that the new *Sticta* species described here have only been found in single localities, suggesting their putative endemism. Previously, probably endemic *Sticta* species were described from Bolivia by Ossowska et al. (2022a), so if all molecularly confirmed species of *Sticta* from Bolivia are considered, 38% of them are endemic. The occurrence of endemic species of *Sticta* has been noted, for example, in Madagascar and the Mascarenes (Simon et al. 2018), as well as in Puerto Rico (Mercado-Díaz et al. 2020). However, as the genus is still not well studied in many regions, some species may appear more widespread, for example, in this paper, we report the first record of *S.riparia* from Bolivia, which has been known only from Puerto Rico so far (Mercado-Díaz et al. 2020).

The question remains open also in the case of how many species occur in Bolivia. The number of Bolivian specimens awaiting revision is still large and taking into account all data from neighbouring countries, we estimate that around 90 species of *Sticta* may occur in Bolivia.

## ﻿Taxonomy

### ﻿Species new to science described from Bolivia

#### ﻿ Sticta isidiolobulata

Taxon classificationFungiPeltigeralesLobariaceae

Ossowska, B. Moncada, Lücking & Kukwa sp. nov.

ECAE2A86-AC04-5859-8DBA-D311EE55684B

852904

Fig. 2

##### Diagnosis.

Differing from *S.impressula* in the presence of isidia developing into spathulate lobules and apothecia with entire to weakly-crenate margins and the presence of sparse, secondary tomentum.

##### Type.

Bolivia. Dept. Cochabamba; Prov. Carrasco, Parque Nacional Carrasco, between Meruvia and Monte Punku, 17°34'43"S, 65°15'25"W, elev. 3082 m, *Podocarpus* forest, Ceja de Monte Inferior (Altimontano), corticolous, 26 Nov. 2014, M. Kukwa 15054 (holotype UGDA, isotype LPB).

##### Description.

Primary photobiont cyanobacterial (*Nostoc*). Stipe absent. Thallus irregular to suborbicular, subcoriaceous, up to 15 cm diam., moderately branched, with 3–5 branches per 5 cm radius, branching polytomous to anisotomic; lobes ligulate to flabellate, adjacent, plane to involute, with their apices rounded and involute and their margins entire to crenate and not thickened; lobe internodes (2–)3–5(–7) mm long, (3–)6–8(–10) mm broad. Upper surface pitted to rugose-foveolate towards the centre, beige brown with slightly darker apices when dry, shiny; surface glabrous, without papillae and pruina, with orbicular to irregular, scattered, pale beige maculae; marginal cilia absent, but extension of the lower tomentum visible. Apothecia abundant, mostly laminal or submarginal, dispersed or rarely grouped in four, subpedicellate to pedicellate, without pronounced invagination on lower side, up to 2.5 mm diam.; disc orange-brown or yellow (in young apothecia), shiny, concave in young apothecia, convex in older; margin entire to weakly crenate, light brown, not visible from surface view in mature apothecia. Vegetative propagules in the form of flattened and branched isidia developing especially on margins into spathulate lobules, aggregate, branched, horizontal, up to 0.25 mm long and 0.5 mm broad, darker than the thallus, brown grey, shiny. Lower surface undulate and veined, beige to light brown towards the centre; primary tomentum dense, but absent towards the margin, thick, but thinner towards the margin, spongy to fasciculate, soft, beige to brown in older parts; secondary tomentum present, pubescent, sparse. Rhizines absent. Cyphellae 1–20 per cm^2^ towards the thallus centre and 21–40 per cm^2^ towards the margin, scattered, rounded or elongated, urceolate with wide pore to cupuliform, prominent, remaining below the level of the primary tomentum, with the margin erect to raised and involute, cream to brown coloured, with tomentum; pore (0.25–)0.5–0.7 mm diam.; basal membrane scabrid, white. Medulla compact, white. Pycnidia present, immersed.

**Figure 2.**
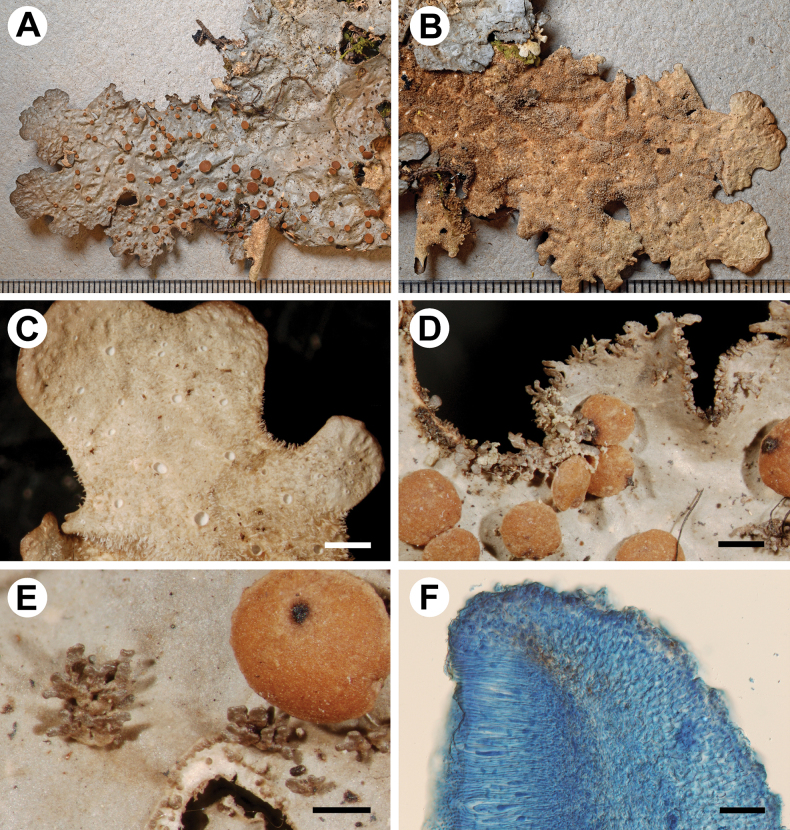
Morphology of *Stictaisidiolobulata* (holotype) **A** upper surface **B** lower surface **C** lower tomentum with cyphellae **D, E** Isidia developing into spathulate lobules and apothecia **F** section of apothecium. Scale bars: 1 mm (**A–D, F, G**); 0.5 mm (**E**); 50 μm (**F**).

Upper cortex paraplectenchymatous, 30–75 μm thick, differentiated into two cellular layers with the upper layer consisting of 1–2 cell layers, cells 4.5–12 × 4.5–7 μm, their walls 1–3.5 μm thick and their lumina rounded to elongated, 4–11 × 3–6 μm. Photobiont layer 25–55 μm thick, its cells 5–10 μm diam. Medulla 50–150 μm thick, its hyphae 2–4 μm broad, without crystals. Lower cortex paraplectenchymatous, 30–60 μm thick, of 2–4 cell layers; cells 6–15 × 6–12 μm diam., their walls 1–3 μm thick. Hairs of lower primary tomentum up to 400 μm long, in fascicles more than 20, hyphae unbranched, septate with free apices; hairs of secondary tomentum 10–18 μm long, 5–6 μm broad, consisting of two 2–4 cells. Cyphellae cavity up to 250 μm deep; cells of basal membrane with many small papillae (up to 0.5 μm high). Apothecia biatorine, up to 500 μm high, without or with distinct stipe; excipulum up to 130 μm broad, without hairs. Hymenium up to 130 μm high; epihymenium 2.5–5 μm high, yellowish, without gelatinous upper layer; epihymenium pale brown-orange. Asci 4–8-spored, ascospores fusiform, 1(–3)-septate, 25–35 × 6–8 μm.

##### Secondary chemistry.

No lichen substances detected by TLC. All parts of thallus and apothecia K–, C–, KC–, P–.

##### Habitat and distribution.

*Stictaisidiolobulata* is known only from the type locality in the Parque Nacional Carrasco in the Cochabamba Department. It was found on tree bark in *Podocarpus* forest.

##### Etymology.

The epithet refers to the presence of isidia that develop into spathulate lobules, especially at the lobe margins.

##### Notes.

*Stictaisidiolobulata* is another morph within the *S.impressula* morphodeme, like *S.pseudoimpressula* and the undescribed ‘*S.isidioimpressula*’ (Moncada 2012; Ossowska et al. 2022a). However, the new species is the only one in this group characterised by the presence of both vegetative propagules and apothecia, isidia developing into spathulate lobules, without true cilia and with beige to brown primary tomentum, which is dense, but absent at the margins. *Stictapseudoimpressula* lacks vegetative propagules, the tomentum is greyish-brown to black and dense at the margins (Ossowska et al. 2022a). In contrast, ‘*S.isidioimpressula*’ produces laminal, white to grey, cylindrical isidia, instead of marginal, greyish-green and spathulate lobules observed in *S.isidiolobulata*. Furthermore, the primary tomentum in *S.isidioimpressula* is dense and sparse towards the margins and without secondary tomentum (Moncada 2012).

The presence of propagules in the form of isidia and lobules is also characteristic of *S.macrofuliginosa* B. Moncada & Lücking from Colombia (Moncada et al. 2015) and *S.parvilobata* Merc.-Díaz from Puerto Rico (Mercado-Díaz et al. 2020). However, in *S.macrofuliginosa*, isidia are cylindrical, whereas in *S.parvilobata*, they are granular to globular. In contrast, the lobules in both species are lobuliform. These taxa also differ from *S.isidiolobulata* in the upper surface of the thallus, which is scrobiculate to foveolate with sparse laminal apothecia in the Colombian species and smooth to scrobiculate without apothecia in the Puerto Rican species. In addition, the primary tomentum is dense to the lobe margins and spongy in *S.macrofuliginosa* and sparse, but sometimes dense and hirsute to fasciculate in *S.parvilobata* (Moncada 2012; Moncada et al. 2015; Mercado-Díaz et al. 2020).

The new species is related to the Colombian ‘*S.pseudosylvatica*’ (Fig. 1), which still awaits formal validation. Both taxa differ in the structure of the upper surface, which is smooth to ribbed in ‘*S.pseudosylvatica*’ and pitted to rugose in *S.isidiolobulata*. Furthermore, ‘*S.pseudosylvatica*’ has abundant, laminal isidia and primary tomentum is dense to the margins (Moncada 2012). The abundance of cyphellae also varies between them and, in ‘*S.pseudosylvatica*’, they occur in amounts of 21–40 per cm^2^ towards the centre and 61–100 per cm^2^ towards the margins, while in the new species, there are 1–20 per cm^2^ and 21–40 per cm^2^, respectively (Moncada 2012).

#### ﻿ Sticta macrolobata

Taxon classificationFungiPeltigeralesLobariaceae

Ossowska, B. Moncada, Lücking & Kukwa sp. nov.

F5C9D63C-70F3-5E0A-B022-1A535FF8BE61

852905

Fig. 3

##### Diagnosis.

Differing from *S.laciniata* in cyanobacteria as photobiont, thallus up to 25 cm in diam., broad lobes, verrucous (rarely weakly crenate) to tomentose apothecial margins, which is often ciliate in the lower part, light to dark brown lower surface and cyphellae with elevated margins.

##### Type.

Bolivia. Dept. Santa Cruz; Prov. Florida, Parque Nacional Amboró, above la Yunga Village, senda Los Helechos, 18°03'30"S, 63°54'36"W, elev. 2330 m, Yungas cloud forest, corticolous, 07 June 2011, M. Kukwa 9801 (holotype UGDA, isotype LPB).

##### Description.

Primary photobiont cyanobacterial (*Nostoc*). Stipe absent. Thallus irregular, coriaceous, up to 25 cm diam., moderately branched, with 4–5 branches per 5 cm radius, branching pleurotomous to polytomous; lobes laciniate to flabellate, plane, with their apices orbicular and involute, margins entire, not thickened, with brown marginal line; lobe internodes 7–14 mm long, 7–50 mm broad. Upper surface smooth to shallowly scrobiculate, light brown to brown with darker apices when dry, shiny; surface glabrous, without papillae and pruina, but with irregular, scattered, pale beige maculae; marginal cilia absent, but extensions of the lower tomentum visible. Apothecia abundant to sparse, principally laminal to submarginal, dispersed to aggregated, pedicellate, with pronounced invagination on the lower side, up to 5 mm diam.; disc plane, brown to chestnut-brown, shiny, epruinose to delicately pruinose; margin persistent, verrucous to tomentose, rarely weakly crenate, often ciliate in the lower part, with brown tomentum, abundant in young apothecia, sparse in old ones. Vegetative propagules absent. Lower surface plane to uneven, light towards the margins and dark brown towards the centre; primary tomentum dense, thick, but thinner towards the margin, spongy to fasciculate, golden-brown in young parts to brown in older with lighter tips; secondary tomentum present, pubescent. Rhizines present, irregularly dispersed, fasciculate to barbate, up to 6 mm, dark brown. Cyphellae 1–20 per cm^2^ towards the thallus centre and 41–60 per cm^2^ towards the margin, scattered, rounded to irregular, urceolate with wide pore, erumpent to sessile, remaining below the level of the primary tomentum, with the margin elevated and involute, brown-coloured, without tomentum or with tomentum at the base; pore (0.25–)0.5–1(–1.5) mm diam.; basal membrane scabrid, yellow. Medulla compact, yellow. Pycnidia present, sparse, immersed.

**Figure 3.**
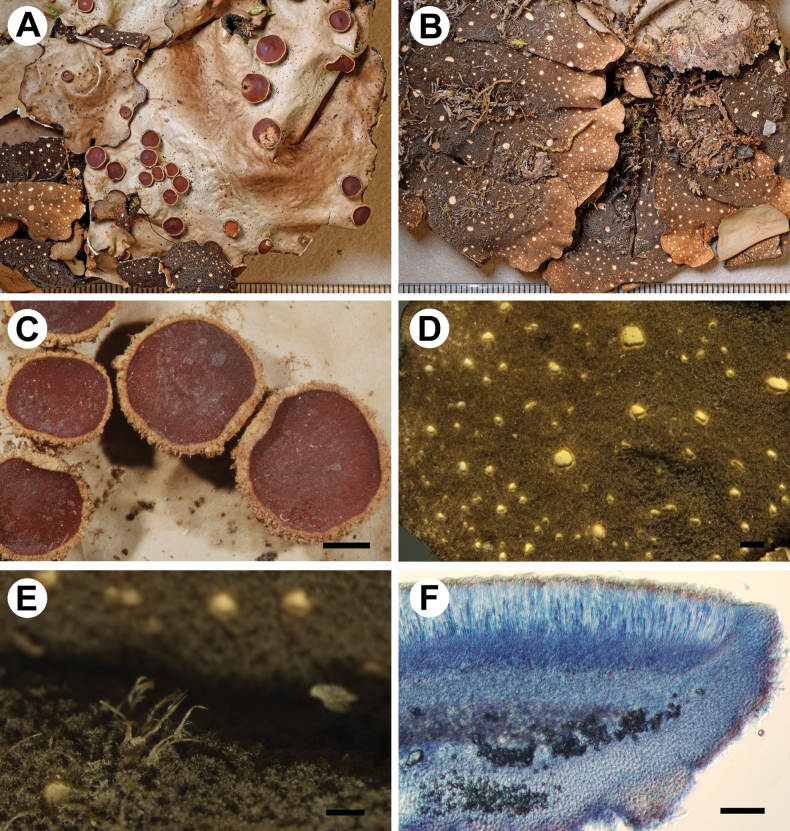
Morphology of *Stictamacrolobata* (holotype) **A** upper surface **B** lower surface **C** apothecia with verrucous to tomentose margins **D** lower tomentum with cyphellae **E** rhizines **F** section of apothecium. Scale bars: 1 mm (**A–E**); 100 μm (**F**).

Upper cortex paraplectenchymatous, 30–40 μm thick, differentiated into two cellular layers with the upper layer consisting of 1–2 layers of small cells, cells 4–15 × 4–10 μm diam., their walls 1–3 μm thick and their lumina rounded to isodiametric, 3–14 μm diam. Photobiont layer 45–75 μm thick, its cells 10–20 μm diam. Medulla 80–120 μm thick, its hyphae 3–4 μm broad. Lower cortex paraplectenchymatous, 30–40 μm thick, homogeneous, consisting of 2–3 layers of cells, cells 7–15 × 6–10 μm, their walls 2–4 μm thick. Hairs of lower primary tomentum up to 220 μm long, in fascicles of more than 20, hyphae simple or rarely branched, 6–8 μm wide with uneven walls, septate with free apices; secondary tomentum sparse, locally developed, up to 2 cells and up to 10 μm long. Cyphellae cavity up to 250 μm deep; cells of basal membrane without papillae. Apothecia biatorine, up to 1 mm high, with distinct stipe; excipulum up to 150 μm broad, laterally with projecting hairs. Hymenium up to 125 μm high; epihymenium up to 10 μm high, brown-orange, with gelatinous upper layer, covered by tiny granules. Asci 6–8-spored, ascospores fusiform, 1(–3)-septate, 25–38 × 6–8 μm.

##### Secondary chemistry.

Unidentified substance in Rf classes A2–3 and C2. Basal membrane of cyphellae K– to K+ pale yellow, C–, KC–, P–. Medulla K+ ochraceous-yellow, C–, KC–, P–.

##### Habitat and distribution.

*Stictamacrolobata* was found on tree bark in Yungas forest. It was collected from a single locality in the Parque Nacional Amboró in the Santa Cruz Department.

##### Etymology.

The name refers to the presence of wide lobes, which are up to 50 mm broad.

##### Notes.

*Stictamacrolobata* resembles *S.laciniata*, but the latter has green photobiont and the thallus is smaller, up to 10 cm broad and more branched than in the new species (Hooker 1822; Moncada 2012). Both species have apothecia with tomentose margins, but in the new species, the margins are also verrucous to rarely weakly crenate and often ciliate in the lower part, whereas in *S.laciniata*, only tomentose. In addition, in *S.macrolobata*, the apothecial discs are brown to chestnut-brown and in *S.laciniata*, orange to reddish (Hooker 1822; Moncada 2012).

The new species forms a clade with *Stictaborinquensis* Merc.-Díaz & Lücking, *S.densiphyllidiata* Merc.-Díaz & Lücking, *S.riparia* and *S.scabrosa* (Fig. 1), although with low support. All four species produce abundant propagules in the form of phyllidia which are absent in the new species (Mercado-Díaz et al. 2020; Moncada et al. 2021b). *Stictaborinquensis* and *S.densiphyllidiata* are epiphytic species known so far from Puerto-Rico (Mercado-Díaz et al. 2020) and *S.riparia* is reported here as new to Bolivia (see below). Stictascabrosasubsp.scabrosa was recently confirmed from Bolivia (Ossowska et al. 2022b) and apothecia were observed in the Bolivian specimens for the first time.

#### ﻿ Sticta madidiensis

Taxon classificationFungiPeltigeralesLobariaceae

Ossowska, B. Moncada, Lücking & Kukwa sp. nov.

B2268975-6983-5122-B7AE-D2EDE3515D82

852906

Fig. 4

##### Diagnosis.

Differing from other *Sticta* in having up to 1 cm long stipe, a palmate to irregular thalli, without vegetative propagules and apothecia, with scabrid upper surface.

##### Type.

Bolivia. Dept. La Paz; Prov. Franz Tamayo, Parque Nacional y Área Natural de Manejo Integrado Madidi, below Keara Bajo, 14°41'90"S, 69°03'51"W, elev. 3060 m, open area with shrubs and scattered trees, Ceja de Monte Inferior (Altimontano), on shrubs, 18 Nov 2014, M. Kukwa 14879 (holotype UGDA, isotype LPB).

##### Description.

Primary photobiont cyanobacterial (*Nostoc*). Stipe present, up to 1 cm long. Thallus palmate to irregular, coriaceous, up to 15 cm diam., moderately branched, with 3–5 branches per 5 cm radius, branching pleurotomous to polytomous; lobes laciniate to ligulate, imbricate, partly involute, with their apices obtuse and acute and their margins entire to sinuous, thickened; lobe internodes 4(7–)–17(–20) mm long, (5–)8–9(–12) mm broad. Upper surface smooth to slightly canaliculate, brown to brownish-grey in central part of thallus when dry, with darker apices and darker marginal line, shiny; surface slightly scrobiculate to rugose, with papillae in young parts of lobes and without pruina, but with irregular, scattered, beige maculae; marginal cilia sparse to abundant fasciculate, white to brown, up to 1 mm, in some areas extension of the lower tomentum present. Apothecia absent. Vegetative propagules absent. Lower surface smooth, yellow-beige to orange-beige; primary tomentum dense, thick, but thinner towards the margin, fasciculate to spongy, soft, whitish-yellow to dark brown in the centre; secondary tomentum present, sparse, pubescent. Rhizines absent. Cyphellae 1–10 per cm^2^ towards the thallus centre and 21–40 per cm^2^ towards the margin, dispersed, rounded to elongate, urceolate with wide pore, erumpent to prominent, remaining below the level of the primary tomentum, with the margin raised and involute or rarely erect, cream to dark brown-coloured, without tomentum; pore (0.25–)0.5–1(–2) mm diam.; basal membrane scabrid, white. Medulla compact, white. Apothecia not found.

**Figure 4.**
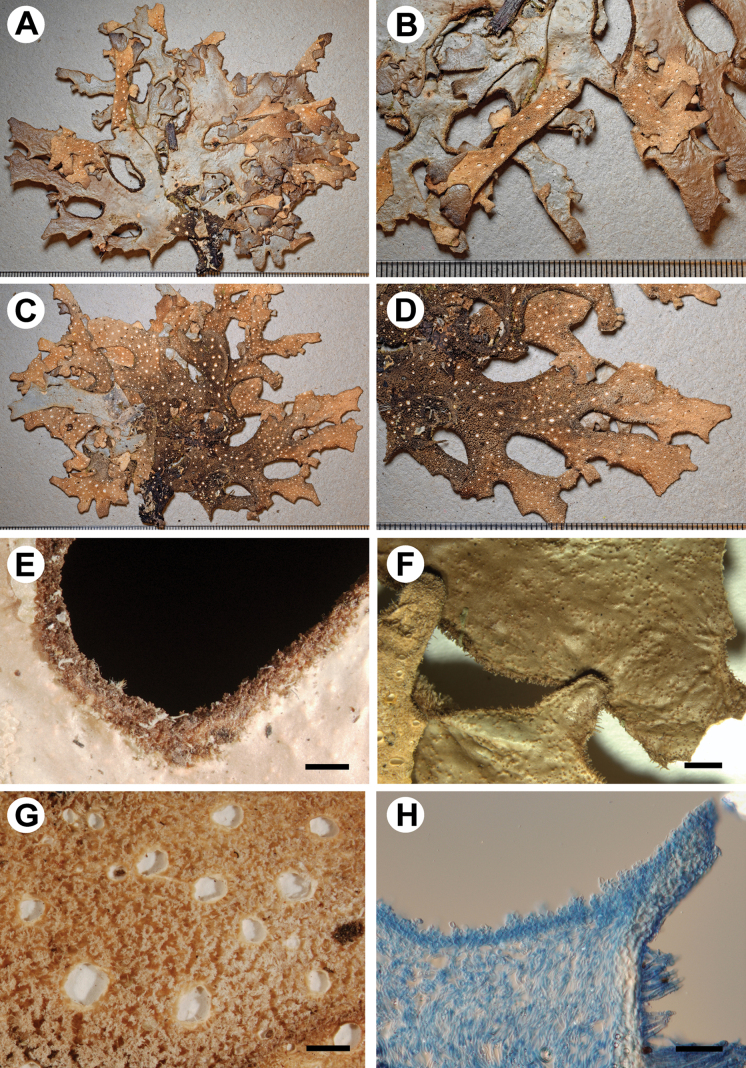
Morphology of *Stictamadidiensis* (holotype) **A, B** upper surface **C, D** lower surface **E** marginal cilia and extension of the lower tomentum **F** scabrid upper surface **G** lower tomentum with cyphellae **H** section of cyphella. Scale bars: 1 mm (**A–G**); 50 μm (**H**).

Upper cortex paraplectenchymatous, up to 50 μm thick, differentiated into two cellular layers with the upper layer consisting of 1–2 layers of smaller cells, cells 5–15 × 5–10 μm, their walls 1–3 μm thick and their lumina rounded to irregular, 4–14 × 4–9 μm. Photobiont layer 30–60 μm thick, its cells 10–20 μm diam. Medulla 110–150 μm thick, its hyphae 4–5 μm broad, without crystals. Lower cortex paraplectenchymatous, 30–40 μm thick, with 2–4 cell layers; cells 7–16 μm × 6–12 diam., their walls 1–3 μm thick. Hairs of lower primary tomentum up to 500 μm long, in fascicles of more than 10, hyphae unbranched, septate with free apices; secondary tomentum sparse of up to 10 μm long. Cyphellae cavity up to 140 μm deep; cells of basal membrane without or with one papilla.

##### Secondary chemistry.

No lichen substances detected by TLC. Basal membrane of cyphellae, K+ yellowish, C–, KC–, P–. Medulla K+ yellowish, C–, KC–, P–.

##### Habitat and distribution.

*Stictamadidiensis* was found on shrubs in mountain vegetation with scattered trees. The species is known only from one locality in in the Madidi protected area in the La Paz Department.

##### Etymology.

The name refers to the type locality.

##### Notes.

The new species has a palmate thallus with a stipe, similar to *S.catharinae* recently described from Bolivia (Ossowska et al. 2022a), which is, however, not related to the new species (Fig. 1). However, cilia in the new species are sparse to abundant and white to brown, whereas in *S.catharinae*, they are abundant, agglutinated to fasciculate, dark brown with paler tips (Ossowska et al. 2022a). Furthermore, *S.catharinae* produces apothecia, which are not known in *S.madidiensis* (Ossowska et al. 2022a). Another taxon with palmate thalli is *S.neopulmonarioides* B. Moncada & Coca (a form with cyanobacteria), but it has abundant, laminal and marginal propagules (phyllidia and lobules) without apothecia (the form with green alga has apothecia, but the thallus is larger than in *S.madidiensis* and irregular). In addition, the primary tomentum is absent towards the margins. *Stictaneopulmonaroides* is known only from Colombia (Moncada 2012; Moncada et al. 2013a).

#### ﻿ Sticta montepunkuensis

Taxon classificationFungiPeltigeralesLobariaceae

Ossowska, B. Moncada, Lücking & Kukwa sp. nov.

846EDEC2-358A-5541-AB81-AA8B8EB3FCBC

852907

Fig. 5

##### Diagnosis.

Differing from other *Sticta* in the green algal photobiont, large thalli up to 30 cm diam., moderately branched, the upper surface scrobiculate to pitted or rugose, the margins of the apothecia crenate to verrucous and the presence of urceolate cyphellae with wide pores and scabrid, white to yellowish-white basal membrane.

##### Type.

Bolivia. Dept. Cochabamba; Prov. Carrasco, Parque Nacional Carrasco, Korikaza close to Monte Punku, 17°33'30"S, 65°16'32"W, elev. 2880 m, lower montane Yungas cloud forest, corticolous, 27 Nov 2014, M. Kukwa 15115 (holotype UGDA, isotype LPB).

##### Description.

Primary photobiont green alga. Stipe absent. Thallus irregular, up to 30 cm diam., moderately branched, with 3–5 branches per 5 cm radius, branching pleurotomous to polytomous; lobes ligulate to laciniate, adjacent to interspaced, plane to involute, with their apices rounded to obtuse and plane and their margins entire, slightly thickened; lobe internodes (7–)10–18(–20) mm long, (5–)10–15(–18) mm broad; thallus coriaceous. Upper surface scrobiculate, pitted or rarely rugose, yellowish-brown and darkening towards the margins when dry, with brown marginal line, shiny; surface glabrous, without papillae, pruina and maculae; marginal cilia absent, but extension of the lower tomentum visible. Apothecia sparse, principally laminal, pedicellate, without pronounced invagination on lower side, up to 0.5 mm diam.; disc brown to red-brown, shiny; margin crenate to verrucous, light cream-brown. Vegetative propagules absent. Lower surface scrobiculate to undulate or faveolate, beige to dark brown towards the centre; primary tomentum dense, thick, but thinner towards the margin, fasciculate, soft, brown often with whitish tips; secondary tomentum present, pubescent to arachnoid. Rhizines sparse, irregularly dispersed, often in groups, fasciculate to barbate, brown with paler tips, up to 1 cm long. Cyphellae 41–60 per cm^2^ towards the thallus centre and more than 100 per cm^2^ towards the margin, scattered, rounded to slightly elongate, urceolate with wide pore, erumpent to sessile, remaining below the level of the primary tomentum, with the margin elevated and involute, brown-coloured, without tomentum; pore (0.3–)0.5–1.8(–2.5) mm diam.; basal membrane scabrid, white to yellowish-white. Medulla compact, white. Pycnidia present.

**Figure 5.**
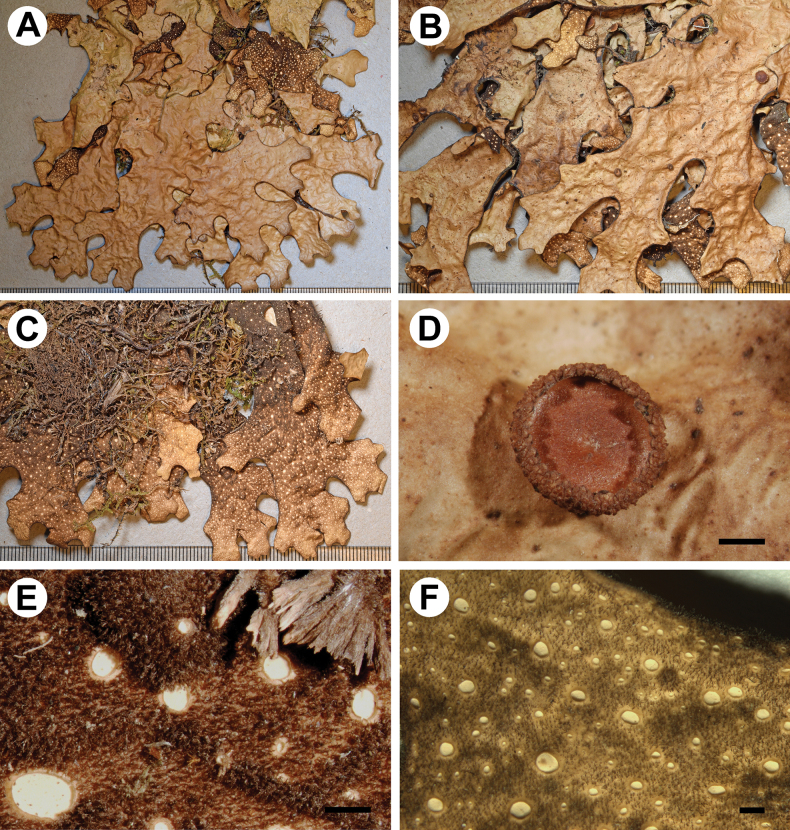
Morphology of *Stictamontepunkuensis* (holotype) **A, B** upper surface **C** lower surface **D** apothecia with crenate to verrucous margins **E, F** lower tomentum with cyphellae and rhizines. Scale bars: 1 mm.

Upper cortex paraplectenchymatous, not distinctly differentiated into layers, 50–65 μm thick, consisting of up to nine cell layers, size of cells gradually decreasing towards the upper part, cells 5–17 × 4–14 μm, their walls 1–4 μm thick and their lumina rounded to isodiametric, 4–16 × 3–13 μm. Photobiont layer 30–50 μm thick, its cells 3.5–6 μm diam. Medulla up to 160 μm thick, its hyphae 3–4.5 μm broad, without crystals. Lower cortex paraplectenchymatous, 35–50 μm thick, with 3–4 cell layers; cells 9–17 × 8–13 μm, their walls 1–3 μm thick. Hairs of lower primary tomentum up to 220 μm long, in fascicles of 6–12, simple or often branched in upper parts, septate with free or interlocked apices, up to 8 μm wide; secondary tomentum up to 25 μm long. Cyphellae cavity up to 100 μm deep; cells of basal membrane usually without or rarely with up to three papillae. Apothecia biatorine, up to 700 μm high, with very short stipe; excipulum up to 250 μm broad, laterally with projecting hairs on the lower side, simple to branched. Hymenium up to 150 μm high; epihymenium up to 10 μm high, pale orange-brown, with gelatinous upper layer, ca. 4 μm high. Asci 6–8-spored, ascospores fusiform, 1(–3)-septate, 17–32 × 7–9 μm.

##### Secondary chemistry.

No lichen substances detected by TLC. All parts of thallus and apothecia K–, C–, KC–, P–.

##### Habitat and distribution.

*Stictamontepunkuensis* is known only from the type locality in Yungas cloud forest in Nacional Parque Carrasco, where it was collected on the bark of tree, at an elevation of 2880 m.

##### Etymology.

The name refers to the settlement Monte Punku in Parque Nacional Carrasco, near where the new species was found.

##### Notes.

The new species is related and morphologically similar to other green algal *Sticta* species, such as *S.lobarioides* and *S.pseudolobaria* (Fig. 1). All these taxa produce apothecia, but they are aggregated, with entire to verrucose margins in *S.lobarioides*, scattered with hairy to verrucous margins in *S.pseudolobaria* and, in *S.montepunkuensis*, they are subaggregated with crenate to verrucous margins. They also differ in the presence of a stipe (absent in *S.montepunkuensis*) and the different sizes of the thalli (up to 20 cm in *S.lobarioides* and over 15 cm in *S.pseudolobaria*). The upper surface in these species is faveolate rather than scrobiculate to pitted as it is in the new species and the primary tomentum is sparse over the entire surface, with no secondary tomentum (primary tomentum dense, secondary present in *S.montepunkuensis*) (Moncada 2012; Moncada et al. 2013a). For the differences between *S.montepunkuensis* and *S.laciniata*, see the general discussion above.

Other species known from Bolivia with green algae and large thalli include *S.amboroensis* and *S.carrascoensis*. The species differ in the structure of the tomentum. In *S.amboroensis*, it is spongy to dense, fasciculate, light to dark brown and sparse towards the margin (Ossowska et al. 2022a). *Stictacarrascoensis* has a primary tomentum that is dense towards the margin like in *S.montepunkuensis*, but it is spongy, light to dark brown, whereas in the new species, it is fasciculate, brown with white tips. *Stictamontepunkuensis* also has more abundant cyphellae, i.e. 41–60 per cm^2^, towards the centre and more than 100 per cm^2^ towards the margin, whereas *S.carrascoensis* has 1–10 per cm^2^ and 21–40 per cm^2^, respectively and *S.amboroensis* 1–20 per cm^2^ and 21–40 per cm^2^ (Ossowska et al. 2022a). Both, *S.amboroensis* and *S.carrascoensis*, have abundant apothecia, which are sparse in the new species and are submarginal in *S.amboroensis* and marginal to laminal in *S.carrascoensis*. Their apothecial margins are crenate to hirsute in both species, rather than crenate to verrucous as in *S.montepunkuensis* (Ossowska et al. 2022a). All three species are not closely related (Fig. 1).

### ﻿Species newly reported from Bolivia

#### ﻿ Sticta beauvoisii

Taxon classificationFungiPeltigeralesLobariaceae

Delise

6A576FAA-A984-53FD-89DA-37BD3792D3CB

Fig. 6

##### Description.

For the description, see McDonald et al. (2003) and Moncada (2012).

##### Habitat and distribution.

The records of *S.beauvoisii* presented here are the first from Bolivia. The species was found on the bark of trees in Tucumano-Boliviano forest at elevations of 1815 m and 1900 m in the Tarija and Chuquisaca Departments. Before, *S.beauvoisii* was known from Colombia and North America: Canada and USA (McDonald et al. 2003; Moncada 2012; Moncada et al. 2020, 2021a).

##### Notes.

*Stictabeauvoisii* is characterised by a smooth, yellowish-brown upper surface with darker apices, without apothecia, but with abundant, marginal, cylindrical to flattened isidia, which are light to dark brown coloured, a brown lower surface, golden-chocolate brown primary tomentum which becomes thin and shorter towards the margins and a sparse, golden-brown, fibrillose to fasciculate rhizines (Delise 1825; McDonald et al. 2003; Moncada 2012).

**Figure 6.**
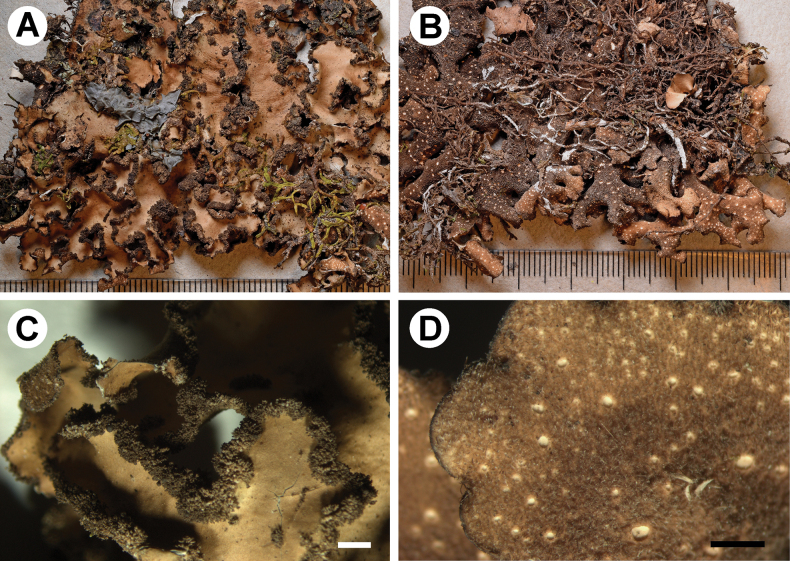
Morphology of *Stictabeauvoisii***A** upper surface (Kukwa 16480) **B** lower surface (Kukwa 16480) **C** marginal isidia (Kukwa 16480) **D** lower surface with tomentum, cyphellae and sparse rhizines (Kukwa 11103). Scale bars: 1 mm.

*Stictabeauvoisii* belongs to clade III sensu Widhelm et al. (2018) (Fig. 1), as do, for example, *S.weigelii* and the undescribed ‘*S.luteocyphellata*’. However, they differ in the colour of the upper and lower surface of the thalli, the isidia and the tomentum. In ‘*S.luteocyphellata*’ the upper surface is light brown, with a brown marginal line and dark brown isidia. The lower surface, on the other hand, is cream to dark brown, with dense in the centre, but sparse towards the margin primary tomentum and greyish-brown with paler apices (Moncada 2012). In *S.weigelii*, the upper surface is reddish-brown to dark brown with a black marginal line and blackish-brown isidia, while the lower surface is beige to reddish-brown with dark brown primary tomentum, dense to the margin (Moncada 2012; Ossowska 2021). Both, ‘*S.luteocyphellata*’ and *S.weigelii* produce abundant rhizines, which are white and fasciculate in ‘*S.luteocyphellata*’ and brownish-black and fibrillose to anziform in *S.weigelii* (Moncada 2012; Ossowska 2021; Torres et al. 2021). In contrast, *S.beauvoisii* has sparse, golden-brown, fibrillose to fasciculate rhizines (McDonald et al. 2003). Another taxon with which *S.beauvoisii* may be confused is the not yet formally described ‘*S.pseudobeauvoisii*’, but it produces narrow phyllidia, rather than isidia and the primary tomentum is light grey to brown, dense and sparse towards the margins (Moncada 2012). ‘*Stictapseudobeauvoisii*’, like *S.beauvoisii*, belongs to clade III of the global *Sticta* phylogeny (Widhelm et al. 2018; Fig. 1).

##### Specimens examined.

Bolivia. Dept. Chuquisaca; Prov. Hernando Siles, 15 km west of Monte Agudo, 19°48'57"S, 64°05'60"W, elev. 1815 m, disturbed Tucumano Boliviano Forest, corticolous, 20 July 2015, M. Kukwa 16480 (LPB, UGDA). Dept. Tarija; Prov. Aniceto Arce, Papachacra, 21°41'52"S, 64°29'15"W, elev. 1900 m, Tucumano Boliviano Forest, corticolous, 8 Aug 2012, M. Kukwa 11103 (LPB, UGDA).

#### ﻿ Sticta riparia

Taxon classificationFungiPeltigeralesLobariaceae

Merc.-Díaz

FC3B9382-EDB5-5388-9CB2-575B1BFDBEE4

Fig. 7

##### Description.

For the description, see Mercado-Díaz et al. (2020).

##### Habitat and distribution.

The record of *S.riparia* presented here is the first one from Bolivia and South America, as the species has been previously known only from Puerto Rico (Mercado-Díaz et al. 2020). In Bolivia, the species was found on tree bark in semi-natural Sub-Andean Amazon forest in the Cochabamba Department.

##### Notes.

*Stictariparia* has a strongly branched thallus, with undulate lobes, the margins of which are covered with branched, abundant, palmate, grey to dark brown phyllidia. The lower surface is greyish-brown, with the primary tomentum absent towards the margins. In addition, cyphellae are abundant, with a density of 41–60 per cm^2^ towards the centre and more than 100 per cm^2^ towards the margins (Mercado-Díaz et al. 2020). It is similar to *S.densiphyllidiata* as, in both species, the lobe margins are abundantly covered by phyllidia, but in *S.densiphyllidiata*, these are dispersed, coralloid and darker than the thallus. Furthermore, the lower surface of the latter taxon is reddish with a dense tomentum. The abundance of the cyphellae towards the margins and centre is also a feature common to both taxa. However, in *S.densiphyllidiata*, the membrane reacts with K+ weakly pink, whereas in *S.riparia*, it is K+ pale yellow (Mercado-Díaz et al. 2020). Both species belong to clade III of the *Sticta* tree (Fig. 1). *Stictadensiphyllidiata* is only known from Puerto Rico (Mercado-Díaz et al. 2020).

Recently, a new phyllidiate species, *S.cerradensis* T.D. Barbosa, J.-M. Torres, Kitaura & A.P. Loren, phylogenetically similar to *S.riparia*, has been described. However, it has larger lobes and the lower surface is light brown to dark. *Stictacerradensis* is only known from Brazil (Torres et al. 2021).

**Figure 7.**
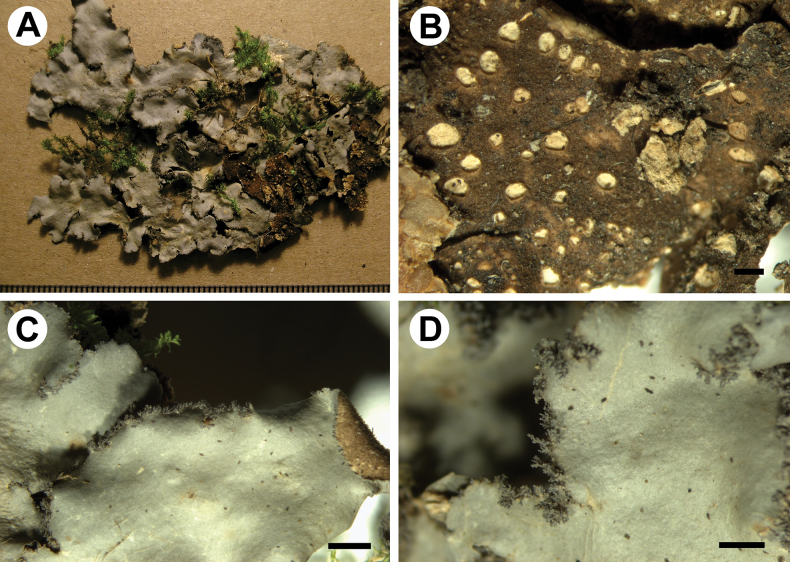
Morphology of *Stictariparia* (Kukwa 18724) **A** upper surface **B** lower surface with very sparse tomentum **C, D** marginal phyllidia. Scale bars: 1 mm.

##### Specimens examined.

Bolivia. Dept. Cochabamba; Prov. Chaparre, Parque Nacional Carrasco, Guacharos, 17°03'50"S, 65°28'31"W, elev. 445 m, semi-natural Sub-Andean Amazon forest, corticolous, 10 Nov 2016, M. Kukwa 18724 (LPB, UGDA).

### ﻿Species confirmed for Bolivia with molecular data

#### ﻿ Sticta tomentosa

Taxon classificationFungiPeltigeralesLobariaceae

(Sw.) Ach.

27229F43-6ADF-5E2D-88C0-2ED542B98AD5

Fig. 8

##### Description.

For a description, see Moncada (2012) and Moncada et al. (2021a).

##### Habitat and distribution.

The record of *S.tomentosa* given here is the first from Bolivia supported by a DNA sequence. The taxon was previously reported from the country by Nylander (1859, 1861) and Herzog (1922). The specimen examined here was found on tree bark in in the lower montane Yungas cloud forest in the Cochabamba Department. Outside Bolivia, *S.tomentosa* has been reported from South and North America (Galloway 1995; Moncada 2012) and Africa (Galloway 1995; Kaasalainen et al. 2023).

##### Notes.

*Stictatomentosa* has palmate, bluish thalli with white cilia, abundant, submarginal apothecia with entire to crenate margins; the lower surface is creamy-white with a sparse, white primary tomentum (Moncada 2012).

The palmate thallus is characteristic for newly-distinguished *S.madidiensis*; however, the taxa differ in the size of the thallus, which is smaller in *S.tomentosa* (up to 5 cm) with abundant, fasciculate cilia. In addition, *S.tomentosa* has abundant apothecia, which are absent in *S.madidiensis*. Both taxa also differ in the structure of the tomentum, which in *S.tomentosa*, is sparse and absent towards the margin and white to greyish-white towards the centre, whereas in *S.madidiensis*, the primary tomentum is dense towards the margin and whitish-yellow to dark brown in the centre (Moncada 2012; Moncada et al. 2021a). Both species are not closely related (Fig. 1).

The species may also be confused with the phylogenetically closely-related *S.leucoblepharis* (Nyl.) Tuck. (Fig. 1), but they differ in the colour of the cilia and the density of the tomentum. In *S.leucoblepharis*, the cilia are golden-brown and longer than in *S.tomentosa*, while the primary tomentum is dense and sparse towards the margins. In addition, the apothecia are laminal rather than submarginal as in *S.tomentosa* and smaller (up to 1.5 cm in diameter) and their discs are orange (Moncada 2012; Moncada et al. 2021a).

Another phylogenetically similar taxon is *S.antoniana* B. Moncada & Lücking and the two cannot be separated, based on nuITS rDNA sequences (Moncada et al. 2020; Moncada et al. 2021a). However, there are important morphological differences. *Stictaantoniana* has an irregular to orbicular and highly-branched thallus, without cilia and with abundant lobules, the primary tomentum is thick and dense, but without secondary tomentum. In *S.tomentosa*, on the other hand, the thallus is palmate to suborbicular, moderately branched, with abundant cilia and without vegetative propagules, while primary tomentum is sparse and absent towards the margin and with secondary tomentum. Both species produce apothecia, but unlike *S.tomentosa*, in *S.antoniana*, they are laminal and with crenate margins (Moncada et al. 2020; Moncada et al. 2021a).

**Figure 8.**
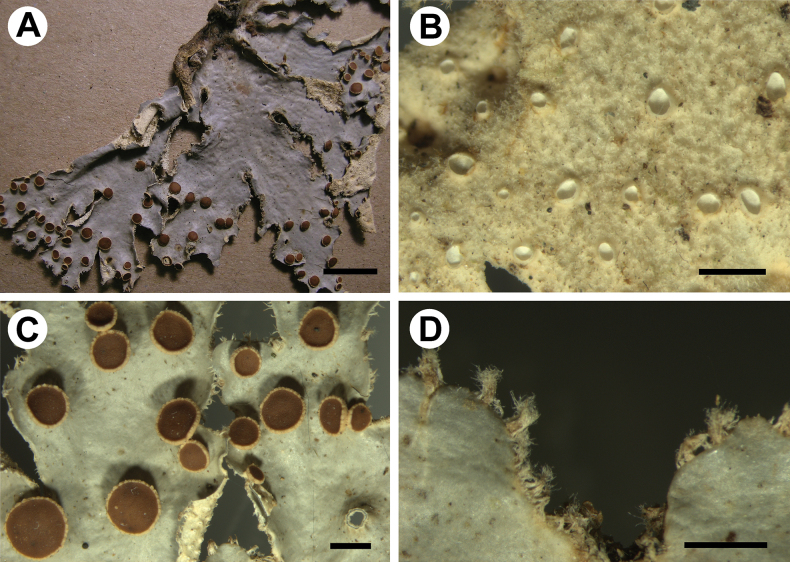
Morphology of *Stictatomentosa* (Kukwa 15138c) **A** upper surface **B** lower surface with tomentum and cyphellae **C** apothecia **D** marginal cilia. Scale bars: 1 cm (**A**); 1 mm (**B–D**).

##### Specimens examined.

Bolivia. Dept. Cochabamba; Prov. Carrasco, Parque Nacional Carrasco, near Rio Ibrisu, close to Sajtarumi, 17°27'09"S, 65°16'29"W, elev. 2059 m, lower montane Yungas cloud forest, corticolous, 28 Nov 2014, M. Kukwa 15138c (LPB, UGDA).

## Supplementary Material

XML Treatment for Sticta isidiolobulata

XML Treatment for Sticta macrolobata

XML Treatment for Sticta madidiensis

XML Treatment for Sticta montepunkuensis

XML Treatment for Sticta beauvoisii

XML Treatment for Sticta riparia

XML Treatment for Sticta tomentosa

## ﻿Additional information

### Author ORCIDs

Emilia Anna Ossowska https://orcid.org/0000-0002-1357-6071

Bibiana Moncada https://orcid.org/0000-0001-9984-2918

Robert Lücking https://orcid.org/0000-0002-3431-4636

Adam Flakus https://orcid.org/0000-0002-0712-0529

Pamela Rodriguez-Flakus https://orcid.org/0000-0001-8300-5613

Martin Kukwa https://orcid.org/0000-0003-1560-909X

### Data availability

All of the data that support the findings of this study are available in the main text or Supplementary Information.

## Supplementary materials

Supplementary material 1 Specimens of *Sticta* used in molecular analysis with locality, voucher information, GenBank accession numbers and list of references Data type docx Explanation note Sequences generated for this study are in bold. https://binary.pensoft.net/file/1031514This dataset is made available under the Open Database License (http://opendatacommons.org/licenses/odbl/1.0/). The Open Database License (ODbL) is a license agreement intended to allow users to freely share, modify, and use this Dataset while maintaining this same freedom for others, provided that the original source and author(s) are credited.Emilia Anna Ossowska, Bibiana Moncada, Robert Lücking, Adam Flakus, Pamela Rodriguez-Flakus, Sandra Olszewska, Martin Kukwa

Supplementary material 2 Best-scoring Maximum Likelihood tree of the *Sticta* target clade containing the new species from Bolivia (red) and the species new to Bolivia and phylogenetically confirmed from Bolivia (blue), based on the fungal ITS barcoding marker Data type pdf Explanation note Supported clades are thickened and individual support values are indicated. https://binary.pensoft.net/file/1031515This dataset is made available under the Open Database License (http://opendatacommons.org/licenses/odbl/1.0/). The Open Database License (ODbL) is a license agreement intended to allow users to freely share, modify, and use this Dataset while maintaining this same freedom for others, provided that the original source and author(s) are credited.Emilia Anna Ossowska, Bibiana Moncada, Robert Lücking, Adam Flakus, Pamela Rodriguez-Flakus, Sandra Olszewska, Martin Kukwa
