# Supplementary material for: ﻿Additional new species and new records of the genus Sticta (lichenised Ascomycota, lobarioid Peltigeraceae) from Bolivia
Source: MycoKeys. 2024 Apr 23;105:21–47. doi: 10.3897/mycokeys.105.120810 (PMC11061559; doi:10.3897/mycokeys.105.120810)
Supplement: Supplementary material 1 — Specimens of Sticta used in molecular analysis with locality, voucher information, GenBank accession numbers and list of references [file mycokeys-105-021-s001.docx]

**Table S1.** Specimens of *Sticta* used in molecular analysis with locality, voucher information, GenBank accession numbers and list of references. Sequences generated for this study are in bold.

| **Species** | **Voucher and references** | **GenBank number** |
| --- | --- | --- |
| *Sticta albocyphellata* | Colombia, MON0246, Moncada et al. (2014) | KC732557 |
| *Sticta albocyphellata* | Colombia, MON0136, Moncada et al. (2014) | KC732520 |
| *Sticta albocyphellata* | Colombia, MON0491, Moncada et al. (2014) | KC732522 |
| *Sticta amboroensis* | Bolivia, Kukwa 9899, Ossowska et al. (2022) | OP250131 |
| *Sticta amboroensis* | Bolivia, Kukwa 9899a, Ossowska et al. (2022) | OP250132 |
| *Sticta andensis* | Colombia, MON0199, Moncada et al. (2014) | KC732547 |
| *Sticta andina* | USA, MON1214, Moncada et al. (2020a) | MT132671 |
| *Sticta andina* | Colombia, MON2182, Moncada et al. (2020b) | MT936705 |
| *Sticta andina* | Mexico, MON4149, Moncada et al. (2020b) | MT936763 |
| *Sticta arachnofuliginosa* | Colombia, MON0113, Moncada et al. (2014) | KC732510 |
| *Sticta arachnofuliginosa* | Colombia, MON0144, Moncada et al. (2014) | KC732525 |
| *Sticta arachnofuliginosa* | Colombia, MON0143, Moncada et al. (2014) | KC732524 |
| *Sticta arachnosylvatica* | Colombia, MON0650, Moncada et al. (2014) | KC732718 |
| *Sticta arachnosylvatica* | Colombia, MON0320, Moncada et al. (2014) | KC732588 |
| *Sticta arachnosylvatica* | Colombia, MON0475, Moncada et al. (2014) | KC732689 |
| *Sticta arbuscula* | Colombia, MON0100c, Moncada et al. (2014) | KC732504 |
| *Sticta arbuscula* | Colombia, MON0100a, Moncada et al. (2014) | KC732502 |
| *Sticta arbuscula* | Colombia, MON0283, Moncada et al. (2014) | KC732569 |
| *Sticta atlantica* | Portugal, LG3858, Magain, Sérusiaux (2015) | KT281737 |
| *Sticta atroandensis* | Colombia, MON0147, Moncada et al. (2014) | KC732528 |
| *Sticta atroandensis* | Colombia, MON0164, Moncada et al. (2014) | KC732532 |
| *Sticta atroandensis* | Colombia, MON0146, Moncada et al. (2014) | KC732527 |
| *Sticta aymara* | Bolivia, Flakus 17220 & Rodriguez-Flakus, Ossowska et al. (2022) | OP250125 |
| *Sticta aymara* | Bolivia, Flakus 17220 & Rodriguez-Flakus, Ossowska et al. (2022) | OP250126 |
| *Sticta beauvoisii* | Bolivia, Kukwa 11103 | **PP273996** |
| *Sticta beauvoisii* | Bolivia, Kukwa 16480 | **PP273995** |
| *Sticta beauvoisii* | Colombia, MON0625, Moncada et al. (2014) | KC732707 |
| *Sticta beauvoisii* | USA, McDonald et al. (2003) | AY173370 |
| *Sticta beauvoisii* | USA, McDonald et al. (2003) | AY173376 |
| *Sticta bicellulata* | Bolivia, Kukwa 14859, Ossowska et al. (2022) | OP250129 |
| *Sticta bicellulata* | Bolivia, Kukwa 14863, Ossowska et al. (2022) | OP250130 |
| *Sticta borinquensis* | Puerto Rico, DNA14658, Mercado-Díaz et al. (2020) | MN065856 |
| *Sticta borinquensis* | Puerto Rico, DNA14660, Mercado-Díaz et al. (2020) | MN065857 |
| *Sticta borinquensis* | Puerto Rico, MON0574, Widhelm et al. (2018) | MG367397 |
| *Sticta brevior* | Colombia, MON0093, Moncada et al. (2014) | KC732499 |
| *Sticta brevior* | Colombia, MON0328, Moncada et al. (2014) | KC732596 |
| *Sticta brevior* | Colombia, MON0175, Moncada et al. (2014) | KC732535 |
| *Sticta canariensis* | Spain, LG1333, Magain, Sérusiaux (2015) | KT281700 |
| *Sticta canariensis* | Ireland, LG3741, Magain, Sérusiaux (2015) | KT281733 |
| *Sticta canariensis* | Spain, Cornejo et al. (2009) | DQ419944 |
| *Sticta carolinensis* | USA, McDonald et al. (2003) | AY173380 |
| *Sticta carolinensis* | USA, McDonald et al. (2003) | AY173381 |
| *Sticta carolinensis* | USA, McDonald et al. (2003) | AY173379 |
| *Sticta carrascoensis* | Bolivia, Kukwa 15028, Ossowska et al. (2022) | OP250133 |
| *Sticta catharinae* | Bolivia, Flakus 17263 & Rodriguez-Flakus, Ossowska et al. (2022) | OP250134 |
| *Sticta catharinae* | Bolivia, Flakus 17263 & Rodriguez-Flakus, Ossowska et al. (2022) | OP250135 |
| *Sticta ciliata* | France, LG3539, Magain, Sérusiaux (2015) | KT281718 |
| *Sticta ciliata* | Ireland, LG3781, Magain, Sérusiaux (2015) | KT281716 |
| *Sticta ciliata* | Spain, LG3099, Magain, Sérusiaux (2015) | KT281715 |
| *Sticta cometia* | Colombia, MON0395, Moncada et al. (2014) | KC732626 |
| *Sticta cometia* | Colombia, MON0412, Moncada et al. (2014) | KC732641 |
| *Sticta cometia* | Colombia, MON0396, Moncada et al. (2014) | KC732627 |
| *Sticta cometiella* | Colombia, MON0339, Moncada et al. (2014) | KC732606 |
| *Sticta cometiella* | Colombia, MON0127, Moncada et al. (2014) | KC732516 |
| *Sticta cometiella* | Colombia, MON0237, Moncada et al. (2014) | KC732556 |
| *Sticta* aff. *cordillerana* | Colombia, MON0225, Moncada et al. (2014) | KC732553 |
| *Sticta densiphyllidiata* | Puerto Rico, MON0575, Widhelm et al. (2018) | MG367398 |
| *Sticta densiphyllidiata* | Puerto Rico, DNA14665, Mercado-Díaz et al. (2020) | MN065890 |
| *Sticta dilatata* | Colombia, MON0419, Moncada et al. (2014) | KC732647 |
| *Sticta dilatata* | Colombia, MON0420, Moncada et al. (2014) | KC732648 |
| *Sticta duplolimbata* | Rwanda, LG0919, Magain, Sérusiaux (2015) | KT281696 |
| *Sticta duplolimbata* | Taiwan | AB245117 |
| *Sticta fragilinata* | USA, McDonald et al. (2003) | AY173385 |
| *Sticta fragilinata* | USA, McDonald et al. (2003) | AY173383 |
| *Sticta fragilinata* | USA, McDonald et al. (2003) | AY173384 |
| *Sticta fuliginoides* | Colombia, MON0806, Widhelm et al. (2018) | MG367410 |
| *Sticta fuliginoides* | UK, LG00S4, Magain, Sérusiaux (2015) | KT281738 |
| *Sticta fuliginoides* | USA, McDonald et al. (2003) | AY173389 |
| *Sticta fuliginosa* | USA, MON1257, Moncada et al. (2020a) | MT132687 |
| *Sticta fuliginosa* | Japan, Takahashi et al. (2006) | AB239345 |
| *Sticta fuliginosa* | Rwanda, LG1611, Magain, Sérusiaux (2015) | KT281702 |
| *Sticta fuscotomentosa* | Colombia, MON0437, Moncada et al. (2014) | KC732661 |
| *Sticta gallowayana* | Colombia, MON0649, Moncada et al. (2014) | KC732717 |
| *Sticta gallowayana* | Colombia, MON0108, Moncada et al. (2014) | KC732507 |
| *Sticta gallowayana* | Colombia, MON0103, Moncada et al. (2014) | KC732496 |
| *Sticta globulifuliginosa* | Colombia, MON0333, Moncada et al. (2014) | KC732601 |
| *Sticta globulifuliginosa* | Colombia, MON0344, Moncada et al. (2014) | KC732608 |
| *Sticta globulifuliginosa* | Colombia, MON4462, Widhelm et al. (2018) | MG754197 |
| *Sticta gyalocarpa* | Colombia, MON0004, Moncada et al. (2014) | KC732455 |
| *Sticta gyalocarpa* | Colombia, MON0326, Moncada et al. (2014) | KC732594 |
| *Sticta gyalocarpa* | Colombia, MON0307, Moncada et al. (2014) | KC732577 |
| *Sticta harrisii* | Puerto Rico, DNA14859, Mercado-Díaz et al. (2020) | MN065831 |
| *Sticta harrisii* | Puerto Rico, MON0576, Moncada et al. (2014) | KC732775 |
| *Sticta harrisii* | Puerto Rico, MON0573, Moncada et al. (2014) | KC732774 |
| *Sticta hirsutofuliginosa* | Colombia, MON0099, Moncada et al. (2014) | KC732501 |
| *Sticta hirsutofuliginosa* | Colombia, MON0347, Moncada et al. (2014) | KC732611 |
| *Sticta hirsutofuliginosa* | Colombia, MON0346, Moncada et al. (2014) | KC732610 |
| *Sticta hirsutogyalocarpa* | Colombia, MON0155, Moncada et al. (2014) | KC732530 |
| *Sticta hirsutogyalocarpa* | Colombia, MON0158, Moncada et al. (2014) | KC732531 |
| *Sticta hirsutogyalocarpa* | Colombia, MON0467, Moncada et al. (2014) | KC732681 |
| *Sticta hirta* | Colombia, MON0117, Moncada et al. (2014) | KC732513 |
| *Sticta hirta* | Colombia, MON0153, Moncada et al. (2014) | KC732563 |
| *Sticta hirta* | Colombia, MON0151, Moncada et al. (2014) | KC732529 |
| *Sticta humboldtii* | Colombia, MON0614, Moncada et al. (2014) | KC732703 |
| *Sticta humboldtii* | Colombia, MON0613, Moncada et al. (2014) | KC732702 |
| *Sticta humboldtii* | Colombia, MON0616, Moncada et al. (2014) | KC732705 |
| *Sticta hypoglabra* | Colombia, MON0455b, Moncada et al. (2014) | KC732668 |
| *Sticta hypoglabra* | Colombia, MON0456b, Moncada et al. (2014) | KC732670 |
| *Sticta hypoglabra* | Colombia, MON0454a, Moncada et al. (2014) | KC732665 |
| *Sticta impressula* | Colombia, MON0418, Moncada et al. (2014) | KC732646 |
| *Sticta impressula* | Colombia, MON0414, Moncada et al. (2014) | KC732643 |
| *Sticta impressula* | Colombia, MON0387, Moncada et al. (2014) | KC732620 |
| *Sticta isidioimpressula* | Colombia, MON0556b, Moncada et al. (2014) | KC732762 |
| *Sticta isidioimpressula* | Colombia, MON0556a, Moncada et al. (2014) | KC732761 |
| *Sticta isidioimpressula* | Colombia, MON0557, Moncada et al. (2014) | KC732763 |
| *Sticta isidiokunthii* | Colombia, MON0324, Moncada et al. (2014) | KC732592 |
| *Sticta isidiokunthii* | Colombia, MON0323, Moncada et al. (2014) | KC732591 |
| *Sticta isidiokunthii* | Colombia, MON0140, Moncada et al. (2014) | KC732523 |
| *Sticta isidiolobulata* | Bolivia, Kukwa 15054 | **PP273989** |
| *Sticta laevis* | Colombia, MON0762, Widhelm et al. (2018) | MG367409 |
| *Sticta latior* | Brazil, MON0273, Moncada et al. (2014) | KC732568 |
| *Sticta leucoblepharis* | Colombia, MON0329, Moncada et al. (2014) | KC732597 |
| *Sticta leucoblepharis* | Colombia, MON0445, Moncada et al. (2014) | KC732664 |
| *Sticta leucoblepharis* | Colombia, MON0330, Moncada et al. (2014) | KC732598 |
| *Sticta limbata* | USA, LG3170, Magain, Sérusiaux (2015) | KT281710 |
| *Sticta limbata* | USA, MON1262, Widhelm et al. (2018) | MG367428 |
| *Sticta limbata* | UK, LG2690, Magain, Sérusiaux (2015) | KT281707 |
| *Sticta lobarioides* | Colombia, MON0236, Moncada et al. (2014) | KC732555 |
| *Sticta lobarioides* | Colombia, MON0404, Moncada et al. (2014) | KC732634 |
| *Sticta lobulata* | Colombia, MON0035, Moncada et al. (2014) | KC732471 |
| *Sticta lobulata* | Colombia, MON0060, Moncada et al. (2014) | KC732482 |
| *Sticta lobulata* | Colombia, MON0496, Moncada et al. (2014) | KC732727 |
| *Sticta lumbschiana* | Colombia, MON0308, Moncada et al. (2014) | KC732578 |
| *Sticta lumbschiana* | Colombia, MON0525, Moncada et al. (2014) | KC732743 |
| *Sticta lumbschiana* | Colombia, MON0305, Moncada et al. (2014) | KC732575 |
| *Sticta macrocyphellata* | Colombia, MON0438, Moncada et al. (2014) | KC732662 |
| *Sticta macrogyalocarpa* | Colombia, MON0518, Moncada et al. (2014) | KC732738 |
| *Sticta macrogyalocarpa* | Colombia, MON0380, Moncada et al. (2014) | KC732619 |
| *Sticta macrolobata* | Bolivia, Kukwa 9801 | **PP273992** |
| *Sticta macrothallina* | Colombia, MON0431b, Moncada et al. (2014) | KC732656 |
| *Sticta macrothallina* | Colombia, MON0431a, Moncada et al. (2014) | KC732655 |
| *Sticta macrothallina* | Colombia, MON0398, Moncada et al. (2014) | KC732629 |
| *Sticta maculofuliginosa* | Colombia, MON0121b, Moncada et al. (2014) | KC732515 |
| *Sticta maculofuliginosa* | Colombia, MON0009, Moncada et al. (2014) | KC732456 |
| *Sticta maculofuliginosa* | Colombia, MON0121a, Moncada et al. (2014) | KC732514 |
| *Sticta madidiensis* | Bolivia, Kukwa 14879 | **PP273990** |
| *Sticta marilandia* | Colombia, MON0550, Moncada et al. (2014) | KC732757 |
| *Sticta marilandia* | Colombia, MON0549, Moncada et al. (2014) | KC732756 |
| *Sticta minutula* | Colombia, MON0315, Moncada et al. (2014) | KC732583 |
| *Sticta minutula* | Colombia, MON0482, Moncada et al. (2014) | KC732719 |
| *Sticta minutula* | Colombia, MON0114, Moncada et al. (2014) | KC732511 |
| *Sticta montepunkuensis* | Bolivia, Kukwa 15115 | **PP273991** |
| *Sticta narinioana* | Colombia, MON2272, Ossowska et al. (2022) | OP244961 |
| *Sticta narinioana* | Colombia, MON2280, Ossowska et al. (2022) | OP244963 |
| *Sticta narinioana* | Colombia, MON2696, Ossowska et al. (2022) | OP244962 |
| *Sticta neopulmonarioides* | Colombia, MON0403, Moncada et al. (2014) | KC732633 |
| *Sticta neopulmonarioides* | Colombia, MON0400, Moncada et al. (2014) | KC732631 |
| *Sticta neopulmonarioides* | Colombia, MON0399, Moncada et al. (2014) | KC732630 |
| *Sticta papillata* | Colombia, MON0832, Widhelm et al. (2018) | MG367414 |
| *Sticta papillata* | Colombia, MON0216, Moncada et al. (2014) | KC732552 |
| *Sticta papillata* | Colombia, MON0215, Moncada et al. (2014) | KC732551 |
| *Sticta paralimbata* | Colombia, MON0182, Moncada et al. (2014) | KC732539 |
| *Sticta paralimbata* | Colombia, MON0030, Moncada et al. (2014) | KC732466 |
| *Sticta pastora* | Colombia, MON0304, Moncada et al. (2014) | KC732574 |
| *Sticta pastora* | Colombia, MON0132, Moncada et al. (2014) | KC732518 |
| *Sticta peltigerella* | Colombia, MON0814, Moncada et al. (2020a) | MT132627 |
| *Sticta peltigerella* | Colombia, MON4410, Moncada et al. (2020a) | MT132763 |
| *Sticta peltigerella* | Colombia, MON0887, Moncada et al. (2020a) | MT132629 |
| *Sticta phyllidiata* | Colombia, MON0046, Moncada et al. (2014) | KC732476 |
| *Sticta phyllidiata* | Colombia, MON0038, Moncada et al. (2014) | KC732558 |
| *Sticta phyllidiata* | Colombia, MON0047, Moncada et al. (2014) | KC732477 |
| *Sticta phyllidiofuliginosa* | Colombia, MON0559, Moncada et al. (2014) | KC732764 |
| *Sticta phyllidiofuliginosa* | Colombia, MON0088, Moncada et al. (2014) | KC732495 |
| *Sticta phyllidiofuliginosa* | Colombia, MON0109, Moncada et al. (2014) | KC732508 |
| *Sticta phyllidiokunthii* | Colombia, MON0534, Moncada et al. (2014) | KC732748 |
| *Sticta phyllidiokunthii* | Colombia, MON0470, Moncada et al. (2014) | KC732684 |
| *Sticta phyllidiokunthii* | Colombia, MON0325, Moncada et al. (2014) | KC732593 |
| *Sticta plumbeociliata* | Colombia, MON0563, Moncada et al. (2014) | KC732767 |
| *Sticta plumbeociliata* | Colombia, MON0560, Moncada et al. (2014) | KC732765 |
| *Sticta psedosylvatica* | Colombia, MON0091, Moncada et al. (2014) | KC732498 |
| *Sticta psedosylvatica* | Colombia, MON0494, Moncada et al. (2014) | KC732725 |
| *Sticta psedosylvatica* | Colombia, MON0493, Moncada et al. (2014) | KC732724 |
| *Sticta pseudohumboldtii* | Colombia, MON0025, Moncada et al. (2014) | KC732463 |
| *Sticta pseudohumboldtii* | Colombia, MON0517, Moncada et al. (2014) | KC732737 |
| *Sticta pseudohumboldtii* | Colombia, MON0515, Moncada et al. (2014) | KC732735 |
| *Sticta pseudoimpressula* | Bolivia, Kukwa 14752, Ossowska et al. (2022) | OP250128 |
| *Sticta pseudoimpressula* | Bolivia, Kukwa 14750, Ossowska et al. (2022) | OP250127 |
| *Sticta pseudolimbata* | Colombia, MON0031, Moncada et al. (2014) | KC732467 |
| *Sticta pseudolimbata* | Colombia, MON0032, Moncada et al. (2014) | KC732468 |
| *Sticta pseudolimbata* | Colombia, MON0185, Moncada et al. (2014) | KC732564 |
| *Sticta pseudolobaria* | Colombia, MON0428, Moncada et al. (2014) | KC732653 |
| *Sticta pseudolobaria* | Colombia, MON0421, Moncada et al. (2014) | KC732649 |
| *Sticta pseudolobaria* | Colombia, MON0425, Moncada et al. (2014) | KC732650 |
| *Sticta pulmonarioides* | Colombia, MON0434, Moncada et al. (2014) | KC732659 |
| *Sticta pulmonarioides* | Colombia, MON0433, Moncada et al. (2014) | KC732658 |
| *Sticta pulmonarioides* | Colombia, MON0402, Moncada et al. (2014) | KC732632 |
| *Sticta puracensis* | Colombia, MON0621, Moncada et al. (2014) | KC732706 |
| *Sticta puracensis* | Colombia, MON0612, Moncada et al. (2014) | KC732701 |
| *Sticta rhizinata* | Colombia, MON0187, Moncada et al. (2014) | KC732542 |
| *Sticta rhizinata* | Colombia, MON0078, Moncada et al. (2014) | KC732491 |
| *Sticta rhizinata* | Colombia, MON0039, Moncada et al. (2014) | KC732559 |
| *Sticta riparia* | Puerto Rico, DNA14513, Mercado-Díaz et al. (2020) | MG367373 |
| *Sticta riparia* | Bolivia, Kukwa 18724 | **PP273994** |
| *Sticta scabrosa* | Costa Rica, MON0906, Moncada et al. (2020b) | MT936648 |
| *Sticta scabrosa* | Brazil, MON5134, Moncada et al. (2020b) | MT936600 |
| *Sticta scabrosa* | Colombia, MON0341, Moncada et al. (2020b) | MT936618 |
| *Sticta squamifera* | Colombia, MON0034, Moncada et al. (2014) | KC732470 |
| *Sticta squamifera* | Colombia, MON0037, Moncada et al. (2014) | KC732473 |
| *Sticta sublimbata* | Japan, Takahashi et al. (2006) | AB245118 |
| *Sticta sublimbata* | Japan, Takahashi et al. (2006) | AB245123 |
| *Sticta sublimbata* | France, LG1038, Magain, Sérusiaux (2015) | KT281699 |
| *Sticta sublimbatoides* | Colombia, MON0647, Moncada et al. (2014) | KC732715 |
| *Sticta sublimbatoides* | Colombia, MON0183, Moncada et al. (2014) | KC732540 |
| *Sticta sublimbatoides* | Colombia, MON0327, Moncada et al. (2014) | KC732595 |
| *Sticta subscrobiculata* | Colombia, MON0204, Moncada et al. (2014) | KC732549 |
| *Sticta subscrobiculata* | Colombia, MON0499, Moncada et al. (2014) | KC732728 |
| *Sticta* aff. *subscrobiculata* | Colombia, MON0512, Moncada et al. (2014) | KC732734 |
| *Sticta* aff. *subscrobiculata* | Colombia, MON0410, Moncada et al. (2014) | KC732639 |
| *Sticta sylvatica* | UK, LG3723, Magain, Sérusiaux (2015) | KT281730 |
| *Sticta sylvatica* | France, LG3536, Magain, Sérusiaux (2015) | KT281726 |
| *Sticta sylvatica* | Ireland, LG3780, Magain, Sérusiaux (2015) | KT281735 |
| *Sticta tomentosa* | Colombia, MON0477, Moncada et al. (2014) | KC732690 |
| *Sticta tomentosa* | Colombia, MON2175, Moncada et al. (2020a) | MT132745 |
| *Sticta tomentosa* | Colombia, MON0442, Moncada et al. (2014) | KC732663 |
| *Sticta tomentosa* | Bolivia, Kukwa 15138c | **PP273993** |
| *Sticta tomentosa* | Ecuador, MON4891, Moncada et al. (2020a) | MT132764 |
| *Sticta viviana* | Colombia, MON0345, Moncada et al. (2014) | KC732609 |
| *Sticta viviana* | Colombia, MON0480, Moncada et al. (2014) | KC732692 |
| *Sticta viviana* | Colombia, MON0462, Moncada et al. (2014) | KC732680 |
| *Sticta weigelii* | Colombia, MON0062, Moncada et al. (2014) | KC732484 |
| *Sticta weigelii* | Puerto Rico, DNA14945, Mercado-Díaz et al. (2020) | MN065896 |
| *Sticta weigelii* | Puerto Rico, DNA14532, Mercado-Díaz et al. (2020) | MN065897 |

**References**

Cornejo C, Chabanenko S, Scheidegger C (2009) Phylogenetic analysis indicates transitions from vegetative to sexual reproduction in the *Lobaria retigera* group (Lecanoromycetidae, Ascomycota). Lichenologist 41 (3): 275–284. https://doi.org/10.1017/S0024282909006240

Magain N, Sérusiaux E (2015) Dismantling the treasured flagship lichen *Sticta fuliginosa* (Peltigerales) into four species in Western Europe. Mycological Progress 14: 97. https://doi.org/10.1007/s11557-015-1109-0

McDonald T, Miadlikowska J, Lutzoni F (2003) The lichen genus *Sticta* in the Great Smoky Mountains: A phylogenetic study of morphological, chemical, and molecular data. The Bryologist 106(1): 61–79. https://doi.org/10.1639/0007-2745(2003)106[0061:TLGSIT]2.0.CO;2

Mercado-Díaz JA, Lücking R, Moncada B, Widhelm TJ, Lumbsch HT (2020) Elucidating species richness in lichen fungi: The genus *Sticta* (Ascomycota: Peltigeraceae) in Puerto Rico. Taxon 69 (5): 1–41. https://doi.org/10.1002/tax.12320

Moncada B, Lücking R, Suárez A (2014) Molecular phylogeny of the genus *Sticta* (lichenized Ascomycota: Lobariaceae) in Colombia. Fungal Diversity 64: 205–231. https://doi.org/10.1007/s1322 5-013-0230-0

Moncada B, Lücking R, Lumbsch HT (2020a) Rewriting the evolutionary history of the lichen genus *Sticta* (Ascomycota: Peltigeraceae subfam. Lobarioideae) in the Hawaiian islands. Plant and Fungal Systematics 65(1): 95–119. https://doi.org/10.35535/pfsyst-2020-0005

Moncada B, Mercado-Díaz JA, Magain N, Hodkinson BP, Smith CW, Bungartz F, Pérez-Pérez R-E, Gumboski E, Sérusiaux E, Lumbsch HT, Lücking R (2020b) Phylogenetic diversity of two geographically overlapping lichens: isolation by distance, environment, or fragmentation? Journal of Biogeography 48(3): 676–689. https://doi.org/10.1111/jbi.14033

Ossowska EA, Moncada B, Kukwa M, Flakus A, Rodriguez-Flakus P, Olszewska S, Lücking R (2022a) New species of *Sticta* (lichenised Ascomycota, lobarioid Peltigeraceae) from Bolivia suggest a high level of endemism in the Central Andes. MycoKeys 92: 131–160. https://doi.org/10.3897/mycokeys.92.89960

Takahashi K, Wang LS, Tsubota H, Deguchi H (2006) Photosymbiodemes *Sticta wrightii* and *Dendriscocaulon* sp. (Lichenized Ascomycota) from Yunnan, China. Journal- Hattori Botanical Laboratory 100: 783–796.

Widhelm TJ, Bertoletti FR, Asztalos MJ, Mercado-Díaz JA, Huang J-P., Moncada B, Lücking R, Magain N, Sérusiaux E, Goffinet B, Crouch N, Mason-Gamer R, Lumbsch HT (2018) Oligocene origin and drivers of diversification in the genus *Sticta* (Lobariaceae, Ascomycota). Molecular Phylogenetics and Evolution 126: 58–73. https://doi.org/10.1016/j.ympev.2018.04.006
