# Supplementary figures and images for: ﻿Additional new species and new records of the genus Sticta (lichenised Ascomycota, lobarioid Peltigeraceae) from Bolivia
Source: MycoKeys. 2024 Apr 23;105:21–47. doi: 10.3897/mycokeys.105.120810 (PMC11061559; doi:10.3897/mycokeys.105.120810)

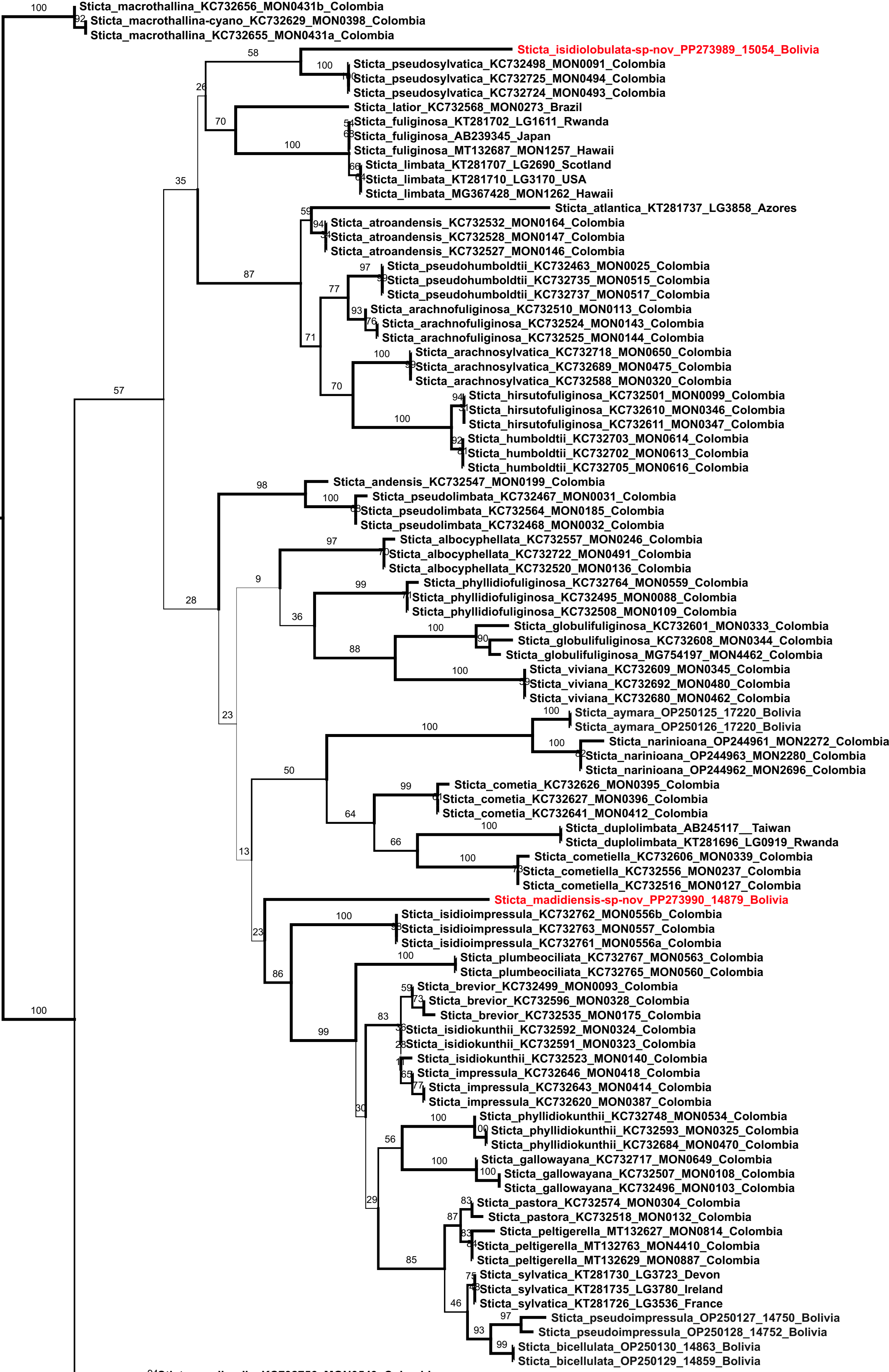

Clade I

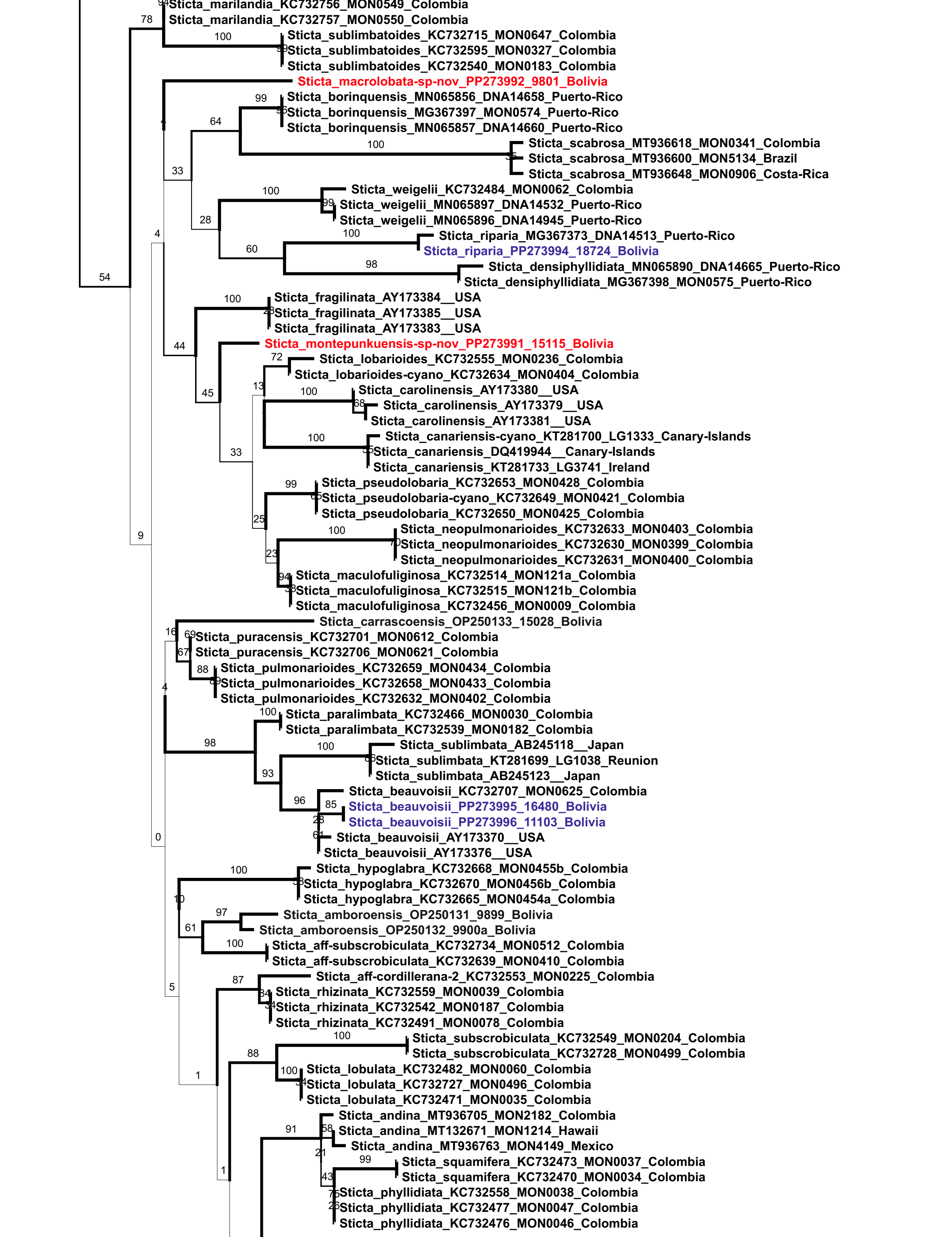

Clade III

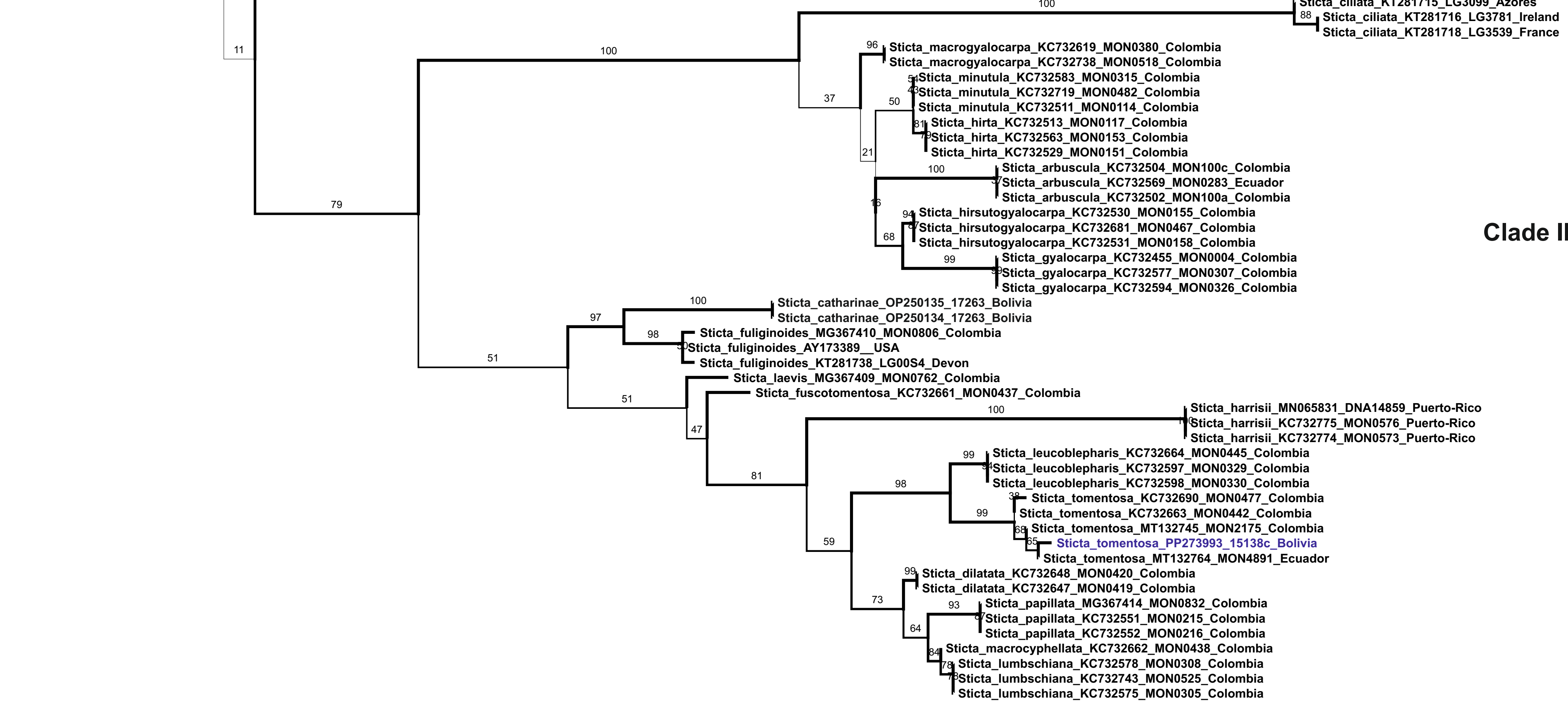

Clade II

Supplement: Supplementary material 2 — Best-scoring Maximum Likelihood tree of the Sticta target clade containing the new species from Bolivia (red) and the species new to Bolivia and phylogenetically confirmed from Bolivia (blue), based on the fungal ITS barcoding marker [file mycokeys-105-021-s002.pdf]
